# Aggressive rat prostate tumors reprogram the benign parts of the prostate and regional lymph nodes prior to metastasis

**DOI:** 10.1371/journal.pone.0176679

**Published:** 2017-05-04

**Authors:** Kerstin Strömvall, Elin Thysell, Sofia Halin Bergström, Anders Bergh

**Affiliations:** Department of Medical Biosciences, Pathology, Umeå University, Umeå, Sweden; University of Pécs Medical School, HUNGARY

## Abstract

In order to grow and spread tumors need to interact with adjacent tissues. We therefore hypothesized that small but aggressive prostate cancers influence the rest of the prostate and regional lymph nodes differently than tumors that are more indolent. Poorly metastatic (Dunning AT1) or highly metastatic (Dunning MLL) rat prostate tumor cells were injected into the ventral prostate lobe of immunocompetent rats. After 10 days—when the tumors occupied about 30% of the prostate lobe and lymph node metastases were undetectable—the global gene expression in tumors, benign parts of the prostate, and regional iliac lymph nodes were examined to define tumor-induced changes related to preparation for future metastasis. The tumors induced profound effects on the gene expression profiles in the benign parts of the prostate and these were strikingly different in the two tumor models. Gene ontology enrichment analysis suggested that tumors with high metastatic capacity were more successful than less metastatic tumors in inducing tumor-promoting changes and suppressing anti-tumor immune responses in the entire prostate. Some of these differences such as altered angiogenesis, nerve density, accumulation of T-cells and macrophages were verified by immunohistochemistry. Gene expression alterations in the regional lymph nodes suggested decreased quantity and activation of immune cells in MLL-lymph nodes that were also verified by immunostaining. In summary, even when small highly metastatic prostate tumors can affect the entire tumor-bearing organ and pre-metastatic lymph nodes differently than less metastatic tumors. When the kinetics of these extratumoral influences (by us named TINT = tumor instructed normal tissue) are more precisely defined they could potentially be used as markers of disease aggressiveness and become therapeutic targets.

## Introduction

In order to grow and spread neoplastic cells need to influence and interact successfully with adjacent cells in the tumor microenvironment and in remote organs such as pre-metastatic niches and the bone marrow [1–3]. For example, malignant prostate cells secrete factors that induce a “reactive tumor stoma” which promotes tumor growth [4, 5]. Prostate cancers also influence major parts of the tumor-bearing organ [6]. The type and magnitude of both the intra- and extratumoral responses are related to tumor aggressiveness [5–8]. In a cohort of prostate cancer patients managed by watchful waiting, quantification of responses in the tumor stroma and in the rest of the tumor-bearing organ could predict prognosis [5, 6, 9–14]. Measuring how the tumor micro- and macro-environments are modified by potentially lethal tumors could therefore be a novel way to evaluate tumor aggressiveness.

We have previously shown that implantation of rat prostate tumor cells into the prostate of immunocompetent syngenic rats resulted in angiogenesis, influx of inflammatory cells, and a gene expression profile similar to a wound healing response in major parts of the tumor-bearing organ [7, 8]. Histological examination showed that small, fast growing and highly metastatic tumors induced more pronounced changes than larger, slow growing and less metastatic tumors [8]. We therefore hypothesized that aggressive cancers, already during early phases of the disease, reprogram large parts of the tumor-bearing organ and probably also other organs differently than tumors that are more indolent [6]. For prostate cancer—a common, multifocal, and highly unpredictable disease—improved diagnostic methods that at an early time point can separate clinically insignificant cancers from potentially lethal cancers are particularly needed [6, 15].

Another site that probably senses the presence of a potentially lethal cancer are the regional lymph nodes (LNs). Experimental studies show that cancers secrete factors preparing selected remote organs to the subsequent arrival of metastatic cells, i.e. they prepare the soil for metastatic growth [16–23]. Such pre-metastatic niches are characterized by a tumor promoting inflammation where myeloid derived suppressor cells and regulatory T-cells prevent anti-tumor immune responses [2, 3, 16, 23–27]. If and how prostate tumors of different malignancy influence regional LNs prior to metastasis, and if so by what mechanisms, are largely unexplored. The few studies available suggest induction of immunosuppressive changes [24, 28–31].

The aim of this study was therefore to define tumor-induced changes in benign parts of the tumor-bearing prostate lobe (in the so-called TINT = tumor instructed normal tissue, see Discussion for details), and in pre-metastatic regional LNs. This was done by exploring the global gene expression profiles in prostate tumor tissue, in prostate TINT, and in pre-metastatic regional LNs from rats carrying either of these two rat prostate tumors: 1) the locally aggressive, poorly differentiated, and low metastatic AT1-tumor, or 2) the locally aggressive, poorly differentiated and highly metastatic MatLyLu (MLL)-tumor (metastasizes primarily to lymph nodes and lung) [32]. We aimed to describe extratumoral changes related to metastatic capacity and explore whether these changes could be linked to tumor-derived signals, either similar signals affecting both the tumor-bearing organ and the regional LNs, or target tissue-specific signals. The long-term goal is to identify novel diagnostic markers and therapeutic targets for subsequent metastatic disease.

## Materials and methods

### Ethics statement

All animal work was carried out in accordance with protocols approved by the Umeå Ethical Committee for animal research (permit number A 42-15A). Adult Copenhagen rats (300–400 g) were housed in a well ventilated room under controlled temperature (25°C), and light (12 h light/ 12 h dark), with food and water available ad libitum. An experienced caretaker monitored the animals on a daily basis. Anesthesia was performed by intraperitoneal injections of Ketamine (75 mg/kg) and Medetomidine (0.5 mg/kg) before tumor cell injection and at sacrifice. Anesthetized animals were euthanatized by removal of the heart. All animals included in the study survived to the endpoint of the experiment and all tumors were small and surrounded by normal prostate tissue, and did not give rise to any symptoms.

### Orthotopic implantation of Dunning R-3327 rat prostate tumor cells

AT1- and MLL- rat prostate tumor cells were purchased from European Collection of Cell Cultures (ECACC, Sigma Aldrich; MLL # 94101454, AT1 # 94101449) and were grown in RPMI 1640 + GlutaMAX (Gibco) supplemented with 10% fetal bovine serum and 250 nM dexamethasone (Sigma Aldrich) [32]. Immunocompetent and syngenic adult Copenhagen rats (Charles River, Sulzfeld, Germany, bred at our animal facility) were used in all experiments. Animals were anesthetized, and an incision was made in the lower abdomen to expose the ventral prostate lobes. 2 x 10^4^ AT1 cells, or 1 x 10^3^ MLL cells (suspended in 10 μl RPMI 1640) were carefully injected into one of the ventral prostate lobes (n = 8 for each tumor type) using a Hamilton syringe with a 30G needle. Rats were sacrificed 10 days after tumor cell injection, the tumor-containing prostates and the regional LNs (iliac nodes) were removed, weighed, frozen in liquid nitrogen, and stored in -80°C. Tissues from untreated rats (n = 8) (and for morphological examination, also from rats injected with vehicle (n = 8)) were used as controls.

### Tissue microdissection, morphology and immunohistochemistry

Ventral prostate tissue was microdissected on cryosections to separate tumor and TINT tissue excluding a border zone of 0.5 mm into the tumor and 0.5 mm into the normal prostate tissue as earlier described [7]. Tumor, prostate TINT and LN cryosections (10 μm, 20–60 sections/tissue type) were used for RNA preparation as described below. The remaining tissue was used for morphological analysis. Maximal tumor- and LN- cross sectional area was measured with the Pannoramic Viewer Software (3DHistech). Cryosections of tumor-, prostate TINT- and LN-tissue were stained with primary antibodies against CD3 (#180102, Invitrogen), CD8 (Clone OX-8, #201701, BioLegend), CD68 (Clone ED1, #MCA341R, Serotec), CD169 (#LS-C124538, LSBio), factor VIII (#A0082, Dako), Ki67 (Clone SP6, #ab16667, Abcam), Lyve-1 (#ab14917, Abcam), and neurofilament-light (NFL, #AB9568, Millipore) to visualize different types of immune cells, blood vessels, proliferating cells, lymph vessels and neurons. “Envision HRP Rabbit” (#K4003, Dako) was used as secondary antibody for CD3, Ki67, factor VIII, NFL, and LYVE1, and “Mach3 Mouse on rat” (#MRT621, Biocare) was used as secondary antibody for CD8, CD68 and CD169. The slides were developed using diaminobenzidine (Dako), except for factor VIII where instead aminoethyl carbazole (Dako) was used. The volume density (percentage of tissue volume occupied by the defined tissue compartment) was determined using point counting morphometry [33]. In brief this was done using a square-lattice mounted in the eyepiece of a light-microscope and counting the number of cross-sections (hits) falling on the immunostained cell type and on the reference tissue space. The calculated values were expressed as means ± standard deviation and the Student’s T-test was used to compare groups using Statistica 12 (Statsoft Inc).

### RNA extraction and cDNA microarray

Total RNA from tumors, prostate TINT, and LNs were extracted using Allprep DNA/RNA/Protein mini kit (Qiagen). The RNA samples (n = 8/group) were sent to the core facility for Bioinformatics and Expression Analysis at Karolinska Institutet (BEA, Novum, KI, Stockholm, Sweden) for RNA integrity evaluation using RNA ScreenTape^®^ and 2200 TapeStation Software (Agilent Technologies), sample preparation and subsequent cDNA microarray analysis on Affymetrix^®^ GeneTitan Gene 1.1 ST Rat array (Affymetrix). Two of the samples from AT1-TINT were excluded due to poor RNA quality.

### Gene expression data analysis

The array data was normalized and pre-processed (Analysis Software = Expression Console, Method = med-polish (rma-bg, quant-norm, sketch = -1, bioc = true, lowprecision = false, usepm = true, target = 0, doavg = false) log2 transformation) at BEA (S1 Dataset). Genes with an average signal intensity > 23 (~4.5 log2) in at least one of the compared groups was included in the analysis. In order to compare groups, average signal intensity of each group was computed as well as fold change (FC) between groups of interest. Student’s T-test was used to test significance of difference between groups. Differentially expressed genes (DEGs) were identified as genes with FC ≥ 1.25 (absolute value) and p-value ≤ 0.05. Venn-diagrams were constructed using Biovenn [34] and Vennplex [35] were used to obtain the numbers of common and unique up-, down-, and contraregulated genes.

The unsupervised multivariate projection method, Principal Component analysis (PCA), was used to build an overview of the sample- and gene variation in the whole-genome cDNA microarray data and to compare subgroups with respect to gene expression and additional characteristics such as LN- and tumor weight (SIMCA 14.0, Umetrics, Umeå, Sweden). Data was mean centered and scaled to unit variance before analysis and all models were validated by cross validation. One tumor and corresponding TINT tissue appeared as apparent outliers in the PCA and was therefore excluded from the study.

QIAGEN’s Ingenuity^®^ pathway analysis (IPA^®^ version 31813283, Qiagen, www.qiagen.com/ingenuity) was used to find potentially affected biological processes represented by the whole-genome cDNA microarray data. Each list of DEGs (9 different comparisons, see below) was analyzed using the IPA core analysis with the IPA default settings. The IPA application returns biological functions or pathways that are overrepresented in the gene expression data. The pathways and functions are given together with a p-value (Fisher’s exact test), and when available, an activation z-score predicting if the particular function or pathway is increased or decreased based on the gene expression pattern and how that correlate to what is reported in the published literature in the manually curated content of the Ingenuity Knowledge Base. A z-score > 2 (absolute value) is considered to be significant. Heatmaps of selected DEGs were made using Multi Experiment Viewer (Mev 4.9.0) software (mev.tm4.org). Due to the large range of signal intensity values, the z-score of each value was computed before data import and heatmap generation.

## Results

### Experimental design

**Fig 1:**
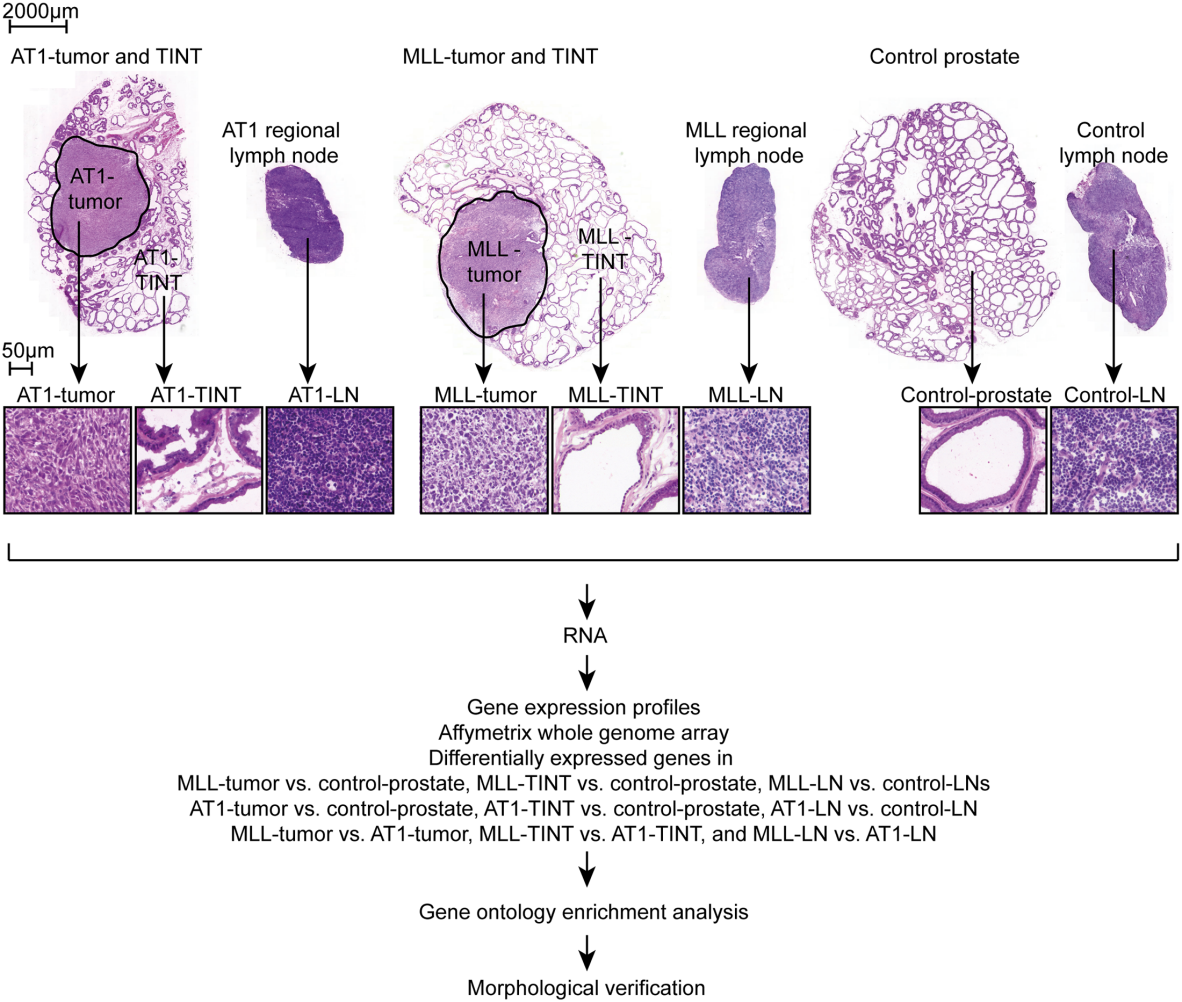
Experimental design. Eosin-hematoxylin stained frozen sections representative of tumors (AT1-tumor and MLL-tumor), prostate TINT (AT1-TINT and MLL-TINT), regional LNs (AT1-LN and MLL-LN), control prostate, and control LNs. The different groups were compared by analyzing global gene expression, gene ontology enrichment analysis, and morphology. TINT, Tumor Instructed Normal Tissue; LN, Lymph Node.

The Dunning R-3327 prostate tumor model consists of several transplantable rat prostate tumor cells lines that were all derived from a spontaneous tumor in the dorsolateral prostate of a Copenhagen rat [32]. The cell lines have different characteristics, thus representing different tumor grades, and can be injected back to fully immunocompetent Copenhagen rats to give tumors *in vivo* [32]. In this study, we used two of these cell lines, AT1 and MatLyLu (MLL). Both these cell lines form tumors *in vivo* that are locally aggressive, poorly differentiated and anaplastic with a poorly differentiated stroma [8, 32] (Fig 1). The main difference between them is that MLL has a much higher ability to metastasize compared to AT1 [32].

AT1 cells (2x10^4^) or MLL cells (1x10^3^) were injected into the prostate of rats and grown for 10 days. At this time point the tumors were of similar sizes (tumor cross-sectional area was 9.6 ± 2.0 mm^2^ for AT1- and 12 ± 4.3 mm^2^ for MLL-tumors, p = 0.26, n = 8 in each group) and were still surrounded by histologically benign normal prostate tissue (the tumors occupied roughly 30% of the prostate lobe volume) (Fig 1). Metastatic tumor cells could not be detected in the regional LNs (Fig 1) or in the lungs (data not shown) using light-microscopy.

Injection of vehicle did not affect the weight of the injected prostate lobe (0.17 ± 0.028 g for vehicle-injected vs. 0.16 ± 0.031 g for untreated animals, p = 0.35, n = 8) and induced very discrete inflammatory changes in the prostate compared to untreated animals, as earlier described [8]. In addition, it did not significantly affect regional LN cross-sectional area measured at the same side as the injection (11 ± 6.0 mm^2^ in vehicle-injected vs. 8.6 ± 2.4 mm^2^ in untreated animals, p = 0.28, n = 6–8). Although we cannot exclude the possibility that surgery and vehicle-injection affect gene expression, this should influence both tumor models similarly and therefore not bias the MLL vs. AT1 comparisons.

Gene expression profiles in AT1- and MLL-tumors and in AT1- and MLL-TINT were compared to profiles in control prostate tissue from treatment naïve animals (Fig 1). Gene expression profiles in AT1- and MLL-LNs were compared to profiles in control LNs from treatment naïve animals (Fig 1). In addition, we compared gene expression profiles in MLL-tumors vs. AT1-tumors, MLL-TINT vs. AT1-TINT and MLL-LNs vs. AT1-LN (Fig 1). To get an overview of the data, the gene expression profiles were analyzed by multivariate analysis and the expression data was summarized with regard to expression intensity and magnitude of changes between groups. Differentially expressed genes (DEGs) were then identified and further analyzed to find associated biological functions using IPA analysis and search tool. Finally, immunohistochemistry was used to verify some of the findings.

### General overview of the whole genome expression data

#### Multivariate analysis

**Fig 2:**
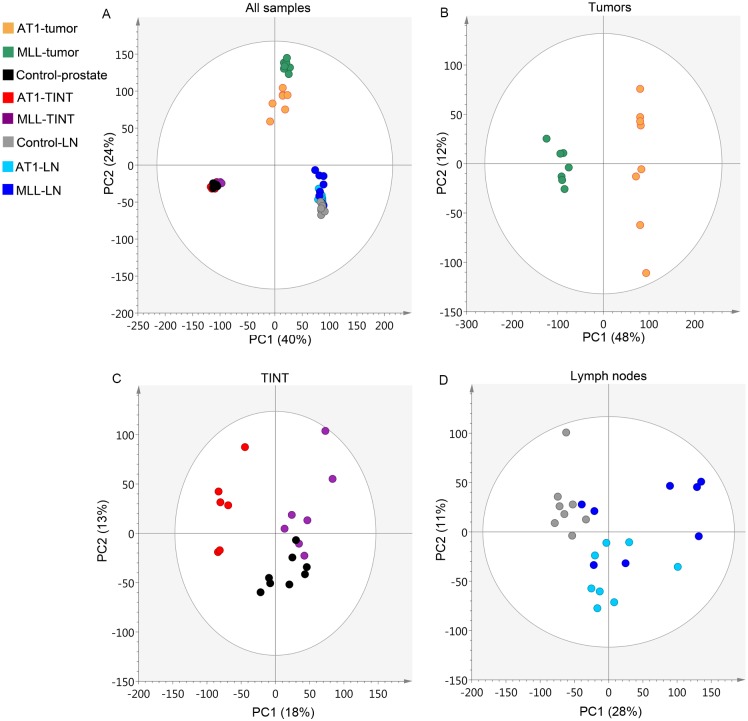
Principal component analysis. Score plots showing the variability explained by the 1^st^ and 2^nd^ components of PCA-models constructed using gene expression signal intensity values in A) all samples, B) tumors, C) prostate TINT and control prostate, and D) regional LNs. MLL-tumor, n = 7; AT1-tumor, n = 8; MLL-TINT, n = 7; AT1-TINT, n = 6; MLL-LN, n = 8; AT1-LN, n = 8; control-prostate, n = 8; control-LN, n = 8. PCA, Principal component analysis; TINT, Tumor Instructed Normal Tissue; LN, Lymph Node.

To get an overview of the gene expression pattern in TINT, and regional LNs induced by highly metastatic MLL-tumors or less metastatic AT1-tumors, we first studied the overall transcriptional profiles by principal component analysis (PCA) of the whole-genome cDNA microarray data. The PCA-score plot show three distinct clusters representing LNs, tumors, and TINT/control-prostate based on unique gene expression profiles (Fig 2A). When examining each tissue individually, the 1^st^ component clearly separated MLL- and AT1-tumors from each other (Fig 2B). In TINT, the 1^st^ component separates AT1-TINT from MLL-TINT and control-prostate (Fig 2C), showing that the gene expression profile in MLL-TINT is more similar to controls than the gene expression profile in AT1-TINT. AT1-LNs and control-LNs formed different clusters although not well separated (Fig 2D) and four of the MLL-LNs formed a separate cluster (Fig 2D). Further examination of the LNs separated by the 1^st^ component showed that this separation was mainly due to LN-size, while the 2^nd^ component separates MLL-LNs from AT1-LNs. Overall, the score plot demonstrates that the gene expression profiles are closely related in the majority of the LNs.

#### Differentially expressed genes

Untransformed signal intensity values, were averaged for each group and used to identify differentially expressed genes (DEGs) between the groups. In tumors, several DEGs had a large fold change (FC) while DEGs in TINT and LNs in general had small FC (S1 Fig). Since both prostate tissue and LNs are composed of multiple cell types, where some populations are small, we considered even a small FC to be of potential importance. For further analysis, we therefore selected DEGs with a FC ≥ 1.25 and a p-value ≤ 0.05. In total we identified 11 445 DEGs in tumors, 1858 DEGs in TINT and 3201 DEGs in LNs (S1C Fig). Overall, the gene expression in MLL- and AT1-tumors was relatively similar, as was the gene expression in the regional LNs from these animals. In contrast, prostate TINT surrounding the two tumor types differed considerably (S1 Fig).

### Gene expression profiles in AT1- and MLL-tumors

#### The most differentially expressed genes in AT1- and MLL-tumors

Although the aim of this study was to characterize extratumoral changes induced by tumors with different metastatic capacity we first wanted to see how the gene expression in two tumor types differed from each other. For this purpose, we listed: 1) top 50 DEGs, 2) top 50 DEGs with a signal intensity value ≥ 500, and 3) top 25 most highly expressed DEGs (S1 Table).

Several of the top genes have well-known biological functions that can probably be related to differences in tumor behavior. Among transcripts with a higher expression in MLL- vs. AT1-tumors we found, for example, *Pi15* –a gene associated with lethal prostate cancer in patients [36, 37], *Fabp4* –a factor associated with bone metastases in prostate cancer patients [38], and *Eya2* –a transcription factor associated with prostate development as well as metastasis in other cancer types [39, 40]. *Dsc3* was markedly lower in MLL- vs. AT1-tumors and reduced expression of this gene has been associated with poor prognosis in prostate cancer patients [41]. LOC100363492 (*Ly6k*), MGC114427 (*Magea9*), and *Mageb1* are known as tumor antigens [42, 43] and were all expressed at a lower level in MLL- than in AT1-tumors, especially *Ly6k*.

#### Gene ontology enrichment analysis of AT1- and MLL-tumors

To further characterize the tumors and find biological functions potentially related to metastasis we used the Ingenuity Pathway Analysis (IPA) analysis and search tool. DEGs with FC ≥ 1.5 and p ≤ 0.05 were included in the analysis. Significant biological functions of interest are shown in S2 Table and the entire result of the downstream effect analysis, Diseases and Functions, are shown in S3 Table. In summary, proliferation of cells appeared increased while chemotaxis, cell movement, and recruitment of cells seemed decreased in MLL- vs. AT1-tumors (S2 Table). Also, many immune-related annotations were predicted to be decreased in MLL- vs. AT-1 tumors, and several annotations related to T-lymphocytes were seen only in AT1-tumors vs. controls (S2 Fig) indicating a more activated (and adaptive) immune response in AT-1 tumors.

#### Morphological differences and verification of selected ontology functions

**Fig 3:**
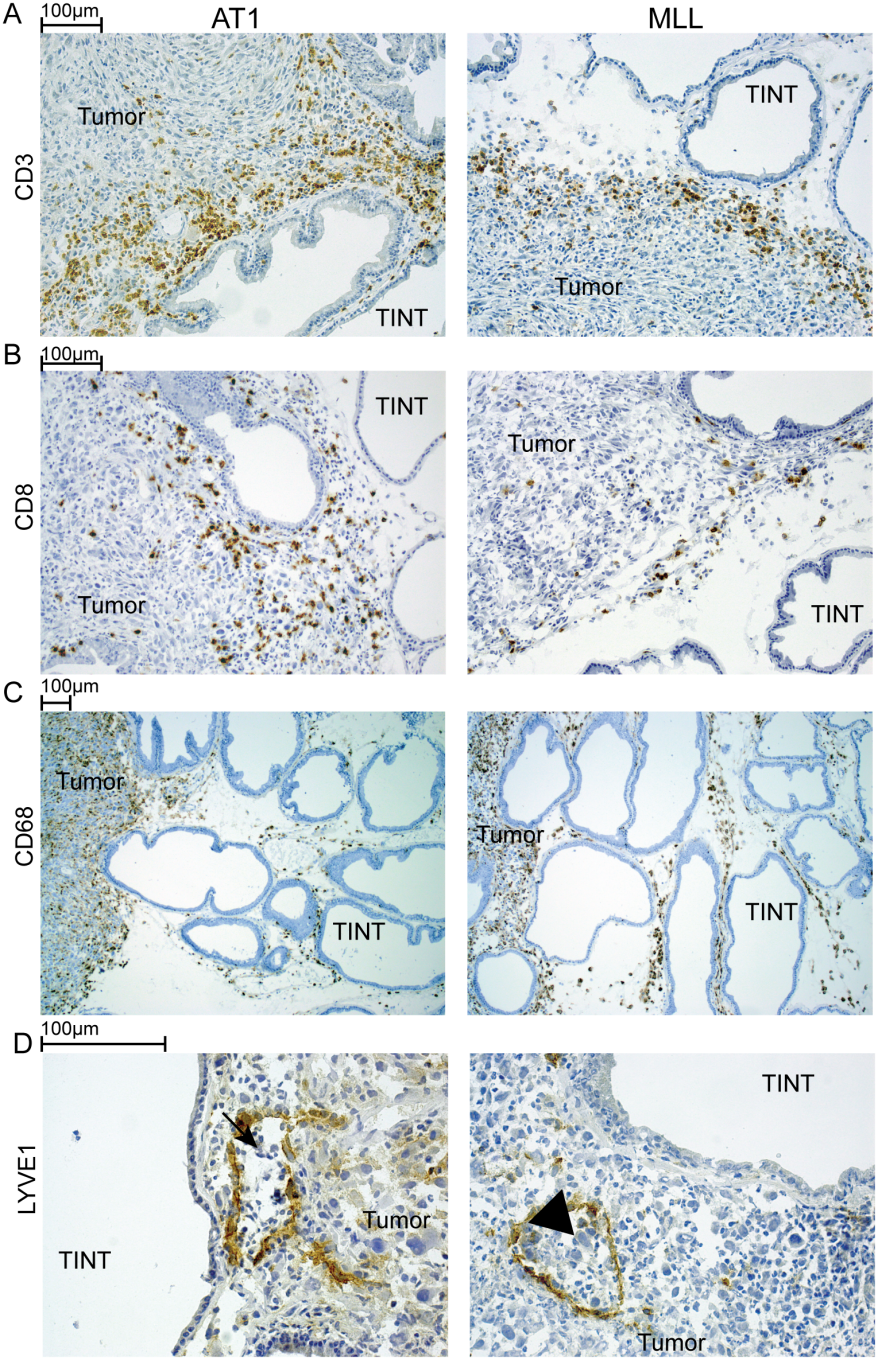
Immunostained tissue sections from AT1- and MLL- tumors. A) CD3^+^ T-lymphocytes (brown) and B) CD8^+^ cells (brown) were more common in the peripheral parts of AT1- than MLL-tumors. In contrast C) CD68^+^ macrophages (brown) were more common in the peripheral parts of MLL-tumors and in MLL-TINT than in the AT1-model. D) Large tumor cells (arrowhead) were seen inside lymph vessels (LYVE1^+^, brown) in the peripheral parts of MLL-tumors. Lymph vessels in AT1-tumors seldom contained tumor cells but more commonly small lymphocyte-like cells (arrow). TINT, Tumor Instructed Normal Tissue.

The Ki67 labeling index appeared higher in MLL- than in AT-1 tumors which is in line with previous quantification [8] (data not shown). The volume density (%) of CD3^+^ intratumoral T-lymphocytes was higher in AT1- than in MLL-tumors (4.4 ± 1.5 vs. 2.5 ± 1.5, p ≤ 0.05, mean ± SD, n = 7) suggesting, consistent with the IPA analysis, increased anti-tumoral immunity in AT1-tumors (Fig 3A). Also, more CD8^+^ cells (presumably cytotoxic T-lymphocytes) were present in the invasive zone of AT1-tumors compared to the invasive zone of MLL-tumors (not quantified, Fig 3B). We have previously shown that MLL-tumors and TINT contain more CD68^+^ macrophages (pan macrophage marker) compared to AT1 [8] and that these macrophages can stimulate tumor growth [44]. In this study, the volume density of CD68^+^ macrophages also appeared higher in MLL- than in AT1-tumors (Fig 3C).

The gene ontology (GO) enrichment analysis in IPA indicated moderately decreased angiogenesis in MLL- vs. AT1-tumors, but the blood vessel volume density (% of tissue composed of factor VIII^+^ vessels) was higher in MLL- than in AT1-tumors (6.6 ± 0.79 vs. 4.4 ± 0.79, p ≤ 0.05, mean ± SD, n = 7). As vessel density was also increased in MLL-TINT (see below), this suggests that blood vessels within tumors may be formed already days earlier as a result of increased angiogenesis in TINT to then be incorporated into the expanding tumor. Very few Lyve1^+^ lymph vessels could be observed within the central parts of the tumors, however, in the peripheral parts, lymph vessels that contained inflammatory cells and tumor cells were sometimes seen (Fig 3D). Tumor cells within the lymph vessels were more common in the peripheral parts of MLL- than in AT1-tumors (Fig 3D).

### Gene expression profiles in AT1- and MLL-TINT

#### The most differentially expressed genes in AT1- and MLL-TINT

To examine differences induced in the tumor-adjacent prostate tissue (TINT) we listed: 1) top 50 DEGs, 2) top 50 DEGs with a signal intensity value ≥ 500, and 3) top 25 most highly expressed DEGs (S4 Table). Several of the genes present in these lists have well-known functions in tumors and in the prostate. Some of the genes most differently expressed in MLL- vs. AT1-TINT are commented below (differences between AT1-TINT vs. control and MLL-TINT vs. control are commented in association with S4 Table).

Top genes upregulated at least 2-fold in MLL- vs. AT1-TINT were for example, *Scl30a2* –an epithelial Zn-transporter shown to be central for prostate function [45], *Grhl3* –a factor that promotes cell migration and invasion [46], *Ubd*–a factor that promotes tumor growth and regulates immune responses [47], *Il12rb2* –an immune regulatory factor [48], *Plau*–a stroma remodeling factor that promotes invasion and metastasis [49], *Clu*—an immune response- and apoptosis regulating factor [50], *Efna5* –a factor suggested to be involved in prostate cancer [51], *Parm1* –an androgen-regulated factor that influence apoptosis in prostate epithelial cells [52], *Edn1* –a factor that promotes prostate cancer growth [53], *Serpine1* –a factor regulating *Plau* (see above, [49]), *Vtcn1* –a factor that restricts antitumor T-cell responses and is associated with metastasis [54], *Mmp15* and *Mmp7* –factors that promote stroma remodeling [55], *Sulf1* –a factor involved in prostate development and cancer [56], *S100a11* –a factor that promotes prostate cancer progression [57], *Ctgf*–a stroma stimulating factor [58], and *Cyr61* –an angiogenic factor that is associated with prostate cancer progression [59].

We further examined if some of the DEGs detected in TINT were regulated in the same way as in the tumors. Of all downregulated genes detected in AT1-TINT 51% were also downregulated in AT1-tumors, and 41% of the upregulated genes were also upregulated in the tumors. In MLL-TINT 69% of the down- and 73% of the upregulated genes were also regulated in the same direction in the MLL-tumors. This suggests that gene expression may be altered in parallel in tumors and in TINT.

#### Gene ontology enrichment analysis of AT1- and MLL-TINT

**Fig 4:**
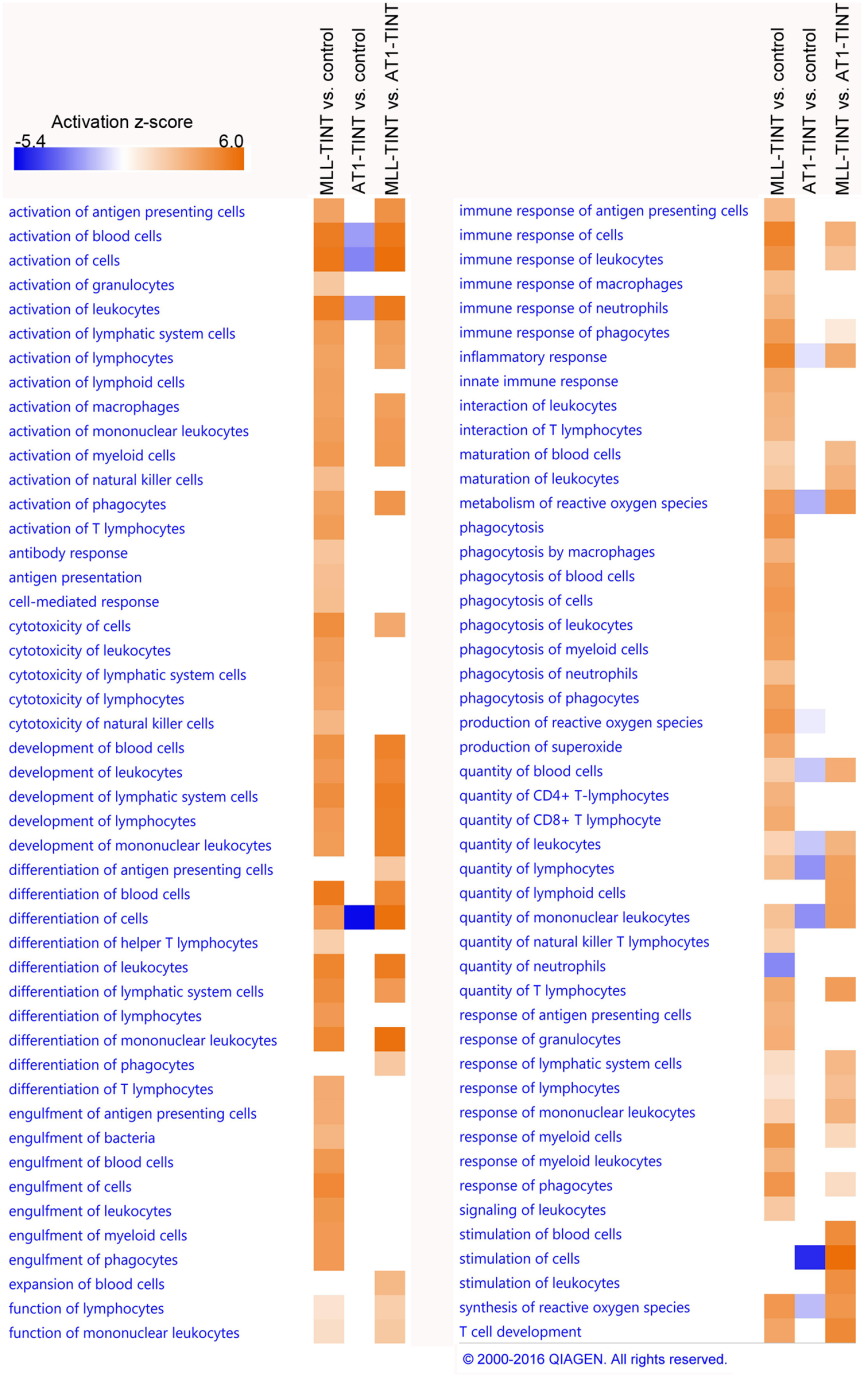
GO enrichment analysis of prostate TINT—Immune-related disease and function annotations. IPA core analyses were performed for each comparison (MLL vs. control, AT1 vs. control, and MLL vs. AT1). DEGs with FC ≥ 1.25 and p ≤ 0.05 were included in the analyses. Significant (p ≤ 0.05, z-score > 2 (absolute value)) immune-related function annotations are shown. The heatmap illustrates the predicted activation z-scores, blue = negative score, decreased activity, and orange = positive score, increased activity. MLL, n = 7; AT1, n = 6; control, n = 8. GO, Gene Ontology; TINT, Tumor Instructed Normal Tissue; IPA, Ingenuity Pathway Analysis; DEG, Differentially Expressed Gene; FC, Fold Change. Reprinted from IPA under a CC BY license, with permission from Qiagen, original copyright 2016.

GO enrichment analysis was performed including all DEGs in TINT with FC ≥ 1.25 and p ≤ 0.05. Significant biological functions of interest are shown in Table 1 (the entire result of the downstream effect analysis, Diseases and Functions, are shown in S5 Table). Several tumor-related biological functions, like invasion, proliferation, tumor growth and angiogenesis, were predicted to be increased in MLL-TINT vs. control-prostate, while decreased in AT1-TINT vs. control-prostate (Table 1). This suggests that MLL-tumors induced a more tumor-promoting environment in the tumor-bearing prostate than AT1-tumors. In addition, several function annotations related to inflammation and recruitment of blood cells were predicted to be increased in MLL-TINT vs. controls and in MLL- vs. AT1-TINT (Table 1, Fig 4). These annotations, if present at all in the analysis of AT1-TINT vs. control-prostate, were predicted to be decreased or had a low activation z-score, suggesting different inflammatory responses in MLL- and AT1-TINT.

**Table 1:** GO enrichment analysis of prostate TINT—Disease and function annotations.

Function annotation	Activation z-score			p-value			# associated genes (% associated genes predicted to increase/decrease/affect function)		
	MLL vs. control	AT1 vs. control	MLL vs. AT1	MLL vs. control	AT1 vs. control	MLL vs. AT1	MLL vs. control	AT1 vs. control	MLL vs. AT1
**Migration of cells**	5.4	-4.4	7.6	4.44E-31	3.18E-20	5.80E-30	183 (66/26/8)	232 (31/60/9)	299 (66/23/11)
**Proliferation of cells**	2.2	-3.1	4.6	9.31E-22	1.31E-14	4.12E-20	254 (49/38/13)	356 (35/51/14)	446 (53/43/5)
**Cell survival**	5.2	-3.4	6.5	6.52E-11	3.26E-05	2.55E-12	107 (66/19/15)	133 (32/57/11)	190 (66/23/11)
**Cell death**	3.0	2.3	-1.3	2.89E-14	4.32E-09	1.29E-13	218 (54/36/10)	308 (52/40/8)	391 (42/49/9)
**Growth of tumor**	2.1	-3.0	3.9	3.49E-14	1.07E-09	4.76E-20	80 (54/34/13)	100 (31/58/11)	145 (59/29/12)
**Invasion of cells**	2.5	-2.7	4.4	6.58E-08	2.04E-12	3.92E-16	69 (59/29/12)	115 (35/57/9)	144 (63/29/8)
**Metastasis**	1.3	-1.8	2.8	3.05E-12	4.18E-12	8.58E-16	75 (35/25/40)	106 (26/38/36)	133 (40/23/37)
**Angiogenesis**	3.1	-3.1	4.4	7.47E-12	2.66E-14	1.02E-18	83 (49/22/29)	126 (25/50/25)	158 (53/23/24)
**Inflammatory response**	4.9	-0.6	3.5	3.91E-35	2.95E-07	5.09E-10	111 (59/19/22)	86 (36/37/27)	110 (53/24/24)
**Chemotaxis of blood cells**	5.3	-	3.5	4.36E-28	-	5.10E-07	64 (67/9/23)	-	52 (62/15/23)
**Homing of blood cells**	5.3	-	3.7	1.10E-27	-	5.07E-07	66 (68/11/21)	-	55 (64/16/20)
**Recruitment of blood cells**	5.0	-1.7	5.0	2.41E-26	1.75E-06	6.54E-10	61 (77/16/7)	43 (33/53/14)	58 (74/14/12)
**Inflammation of organ**	-1.7	3.9	-2.4	4.10E-26	7.51E-07	6.75E-16	130 (29/40/31)	125 (46/18/35)	181 (31/43/26)
**Growth of connective tissue**	0.4	-2.8	3.1	2.41E-07	2.45E-09	1.02E-13	56 (52/41/7)	84 (30/58/12	112 (55/31/13)
**Neuritogenesis**	-	-3.6	3.6	-	6.02E-08	6.40E-07	-	78 (17/46/37)	88 (45/17/38)

IPA core analyses were performed for each comparison (MLL vs. control, AT1 vs. control, and MLL vs. AT1). DEGs with FC ≥ 1.25 and p ≤ 0.05 were included in the analyses. Selected disease and function annotations from the downstream effect analysis are listed in the table. MLL, n = 7; AT1, n = 6; control, n = 8. GO, Gene Ontology; TINT, Tumor Instructed Normal Tissue; IPA, Ingenuity Pathway Analysis; DEG, Differentially Expressed Gene; FC, Fold Change.

**Fig 5:**
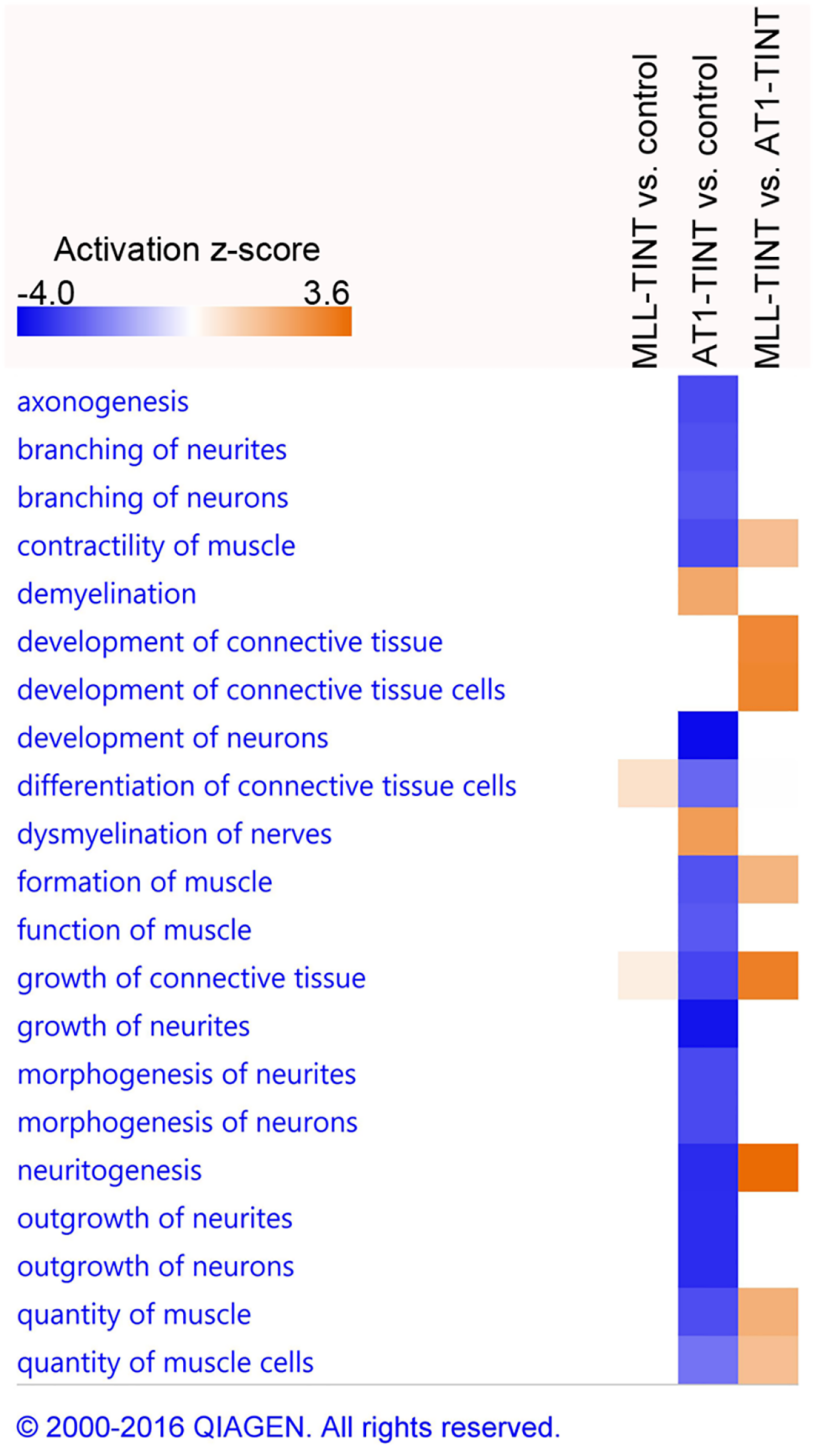
GO enrichment analysis of prostate TINT—Stroma-related disease and function annotations. IPA core analyses were performed for each comparison (MLL vs. control, AT1 vs. control, and MLL vs. AT1). DEGs with FC ≥ 1.25 and p ≤ 0.05 were included in the analyses. Significant (p ≤ 0.05, z-score > 2 (absolute value)) stroma-related function annotations are shown. The heatmap illustrates the predicted activation z-scores, blue = negative score, decreased activity, and orange = positive score, increased activity. MLL, n = 7; AT1, n = 6; control, n = 8. GO, Gene Ontology; TINT, Tumor Instructed Normal Tissue; IPA, Ingenuity Pathway Analysis; DEG, Differentially Expressed Gene; FC, Fold Change. Reprinted from IPA under a CC BY license, with permission from Qiagen, original copyright 2016.

Moreover, annotations related to growth of connective tissue and neuritogenesis were predicted to be decreased in AT1-TINT vs. control-prostate and were not present at all in the analysis of MLL-TINT vs. control prostate (Table 1, Fig 5), indicating that AT1-tumors may have negative effects on stroma fibroblasts and neurons in the tumor-bearing prostate.

**Fig 6:**
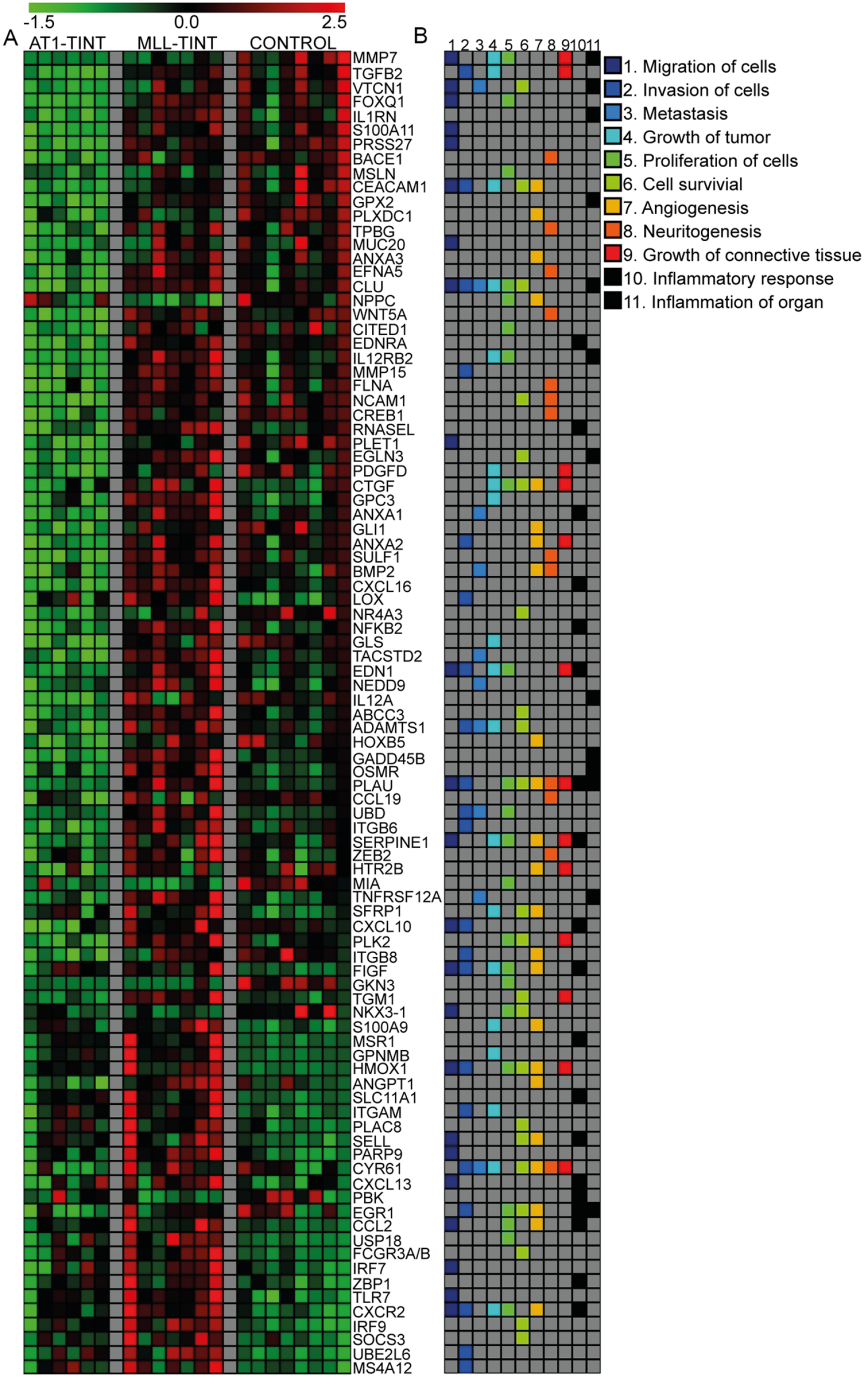
TINT gene expression heatmap. A) Expression of DEGs associated with affected biological functions in TINT, as predicted by the IPA algorithm (Table 1). The top 10 DEGs (highest FC) in support of the prediction made by IPA are included. Top genes present in more than one analysis are presented once. Gene expression signal intensity z-score is showed for each sample, black = mean signal intensity, red = number of standard deviations above the mean, green = number of standard deviations below the mean. B) The multicolored panel shows gene-function associations. MLL, n = 7; AT1, n = 6; control, n = 8. TINT, Tumor Instructed Normal Tissue; DEG, Differentially Expressed Gene; FC, Fold Change; IPA, Ingenuity Pathway Analysis.

To illustrate what genes that might be responsible for the identified functional differences between MLL- and AT1-TINT (Table 1), we specifically searched for genes that were most differentially expressed and at the same time supported the prediction of change made by the IPA algorithm (Fig 6). For example, *Ceacam1*—a factor associated with angiogenesis and tumor aggressiveness in other tumor types [60], *Ctgf*—a factor that stimulates angiogenesis and prostate cancer progression ([58], see above), *Gli1* –a factor that promotes prostate cancer progression [61, 62], *Anxa2*—a factor that is related to prostate cancer metastasis [63], *Bmp2* –a factor that increase prostate cancer motility [64], *Lox*–a factor that influence pre-metastatic niches and is associated with prostate cancer outcome [65, 66], *Figf (Vegfd)*—a lymphangiogenic growth factor [67], *Angpt1*—a factor that promotes blood vessel maturation [68], *Itgam* (*CD11b*)—a factor expressed on leukocytes, *Egr1*—a transcription factor that promotes prostate cancer progression [69], *Ccl2*—a macrophage-attracting factor that promotes prostate cancer growth [70], *Cxcr2*—a cytokine receptor associated with prostate cancer metastasis [71], and already mentioned above *Plau*, *Serpine1*, and *Cyr61*. All these factors had a higher expression in MLL- than in AT1-TINT. Only 7 (*Cxcr2*, *Ednra*, *Flna*, *Il12a*, *Itgb6*, *Prss27*, *Sulf1*) of the 93 DEGs in MLL- vs. AT1-TINT that were related to the functional differences shown in Fig 6 were correlated to tumor size (correlation coefficients 0.55–0.66, p ≤ 0.05, data not shown) suggesting that tumor type could be more important than tumor size for the type of TINT changes induced (see [8] for discussion).

**Fig 7:**
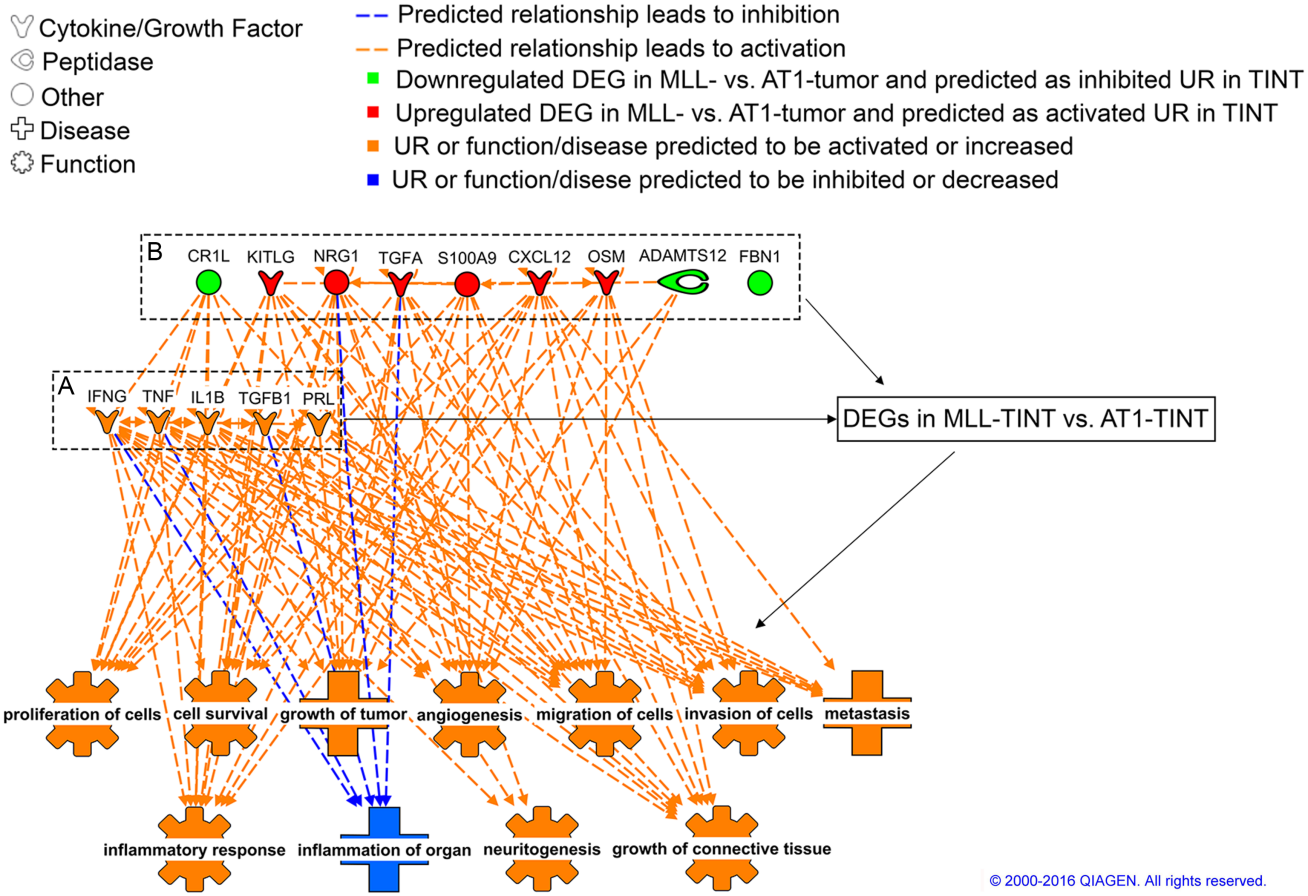
GO enrichment analysis of prostate TINT—Upstream regulators. IPA core analysis of MLL- vs. AT1-TINT DEGs (FC ≥ 1.25 and p ≤ 0.05) was performed. The figure shows predicted upstream regulators. A) the five most significant (according to p-value) upstream regulators overall and B) upstream regulators that could possibly be secreted from the tumors. All upstream regulators are associated with the predicted downstream effects via relations to target genes in TINT (black arrows), but some are also directly associated. UR, Upstream Regulator; GO, Gene Ontology; TINT, Tumor Instructed Normal Tissue; IPA, Ingenuity Pathway Analysis; DEG, Differentially expressed gene; FC, Fold Change. Reprinted from IPA under a CC BY license, with permission from Qiagen, original copyright 2016.

The IPA upstream analysis identified regulators that can explain the observed gene expression changes comparing MLL-TINT with AT1-TINT (S5 Table) and indicated for example IFN-gamma, IL-1B, TGF-beta1, TNF, and prolactin as potential upstream regulators in TINT. Many of these factors are well-known mediators of inflammation and the formation of a reactive tumor stroma [1–5] suggesting that they may be central in the formation of a growth-and metastasis-promoting environment in the tumor-bearing organ (Fig 7A).

Interestingly, among DEGs identified in MLL- vs. AT1-tumors we found some that were suggested to be upstream regulators of the differential gene expression in MLL- vs. AT1-TINT. Genes with higher expression in MLL- vs. AT1-tumors whose gene products potentially could be tumor-derived secreted factors were *Cxcl12* (5-fold increased), *Kitlg* (3-fold), *Osm* (2-fold), *S100a9* (10-fold), and *Tgfa* (2-fold), and genes with higher expression in AT1-tumors than in MLL-tumors were *Adamts12* (8-fold), *Fbn1* (3-fold) and *Cr1l* (2-fold) (Fig 7B). These genes have been associated with functions related with tumor behavior for example, CXCL12 promotes tumor growth locally and in pre-metastatic niches (see [2] for review). Kit-ligand (KITLG) is, by activating c-kit, associated with tumor aggressiveness in other tumor types [72]. S100A9, produced by tumor cells and by infiltrating myeloid suppressor cells, creates a tumor-promoting and immunosuppressive microenvironment in primary tumors and in pre-metastatic niches [2, 27, 73, 74]. In patients, S100A9 is upregulated in aggressive prostate tumors and in the rest of the tumor-bearing prostate [13]. Tumor-derived exosomes carrying miRNAs are known to reprogram other tissues for tumor growth [75], we therefore explored the tumor expression data for differentially expressed miRNAs that could be potential upstream regulators in TINT but no obvious candidates were detected.

#### Morphological differences and verification of selected ontology functions

The volume densities (%) of T-lymphocytes (CD3^+^), lymph vessels (Lyve1^+^), blood vessels (factor VIII^+^), macrophages (CD68^+^) and neurons (NFL^+^) were not significantly different in the prostate tissue of vehicle-injected animals and treatment naïve animals (data not shown).

**Fig 8:**
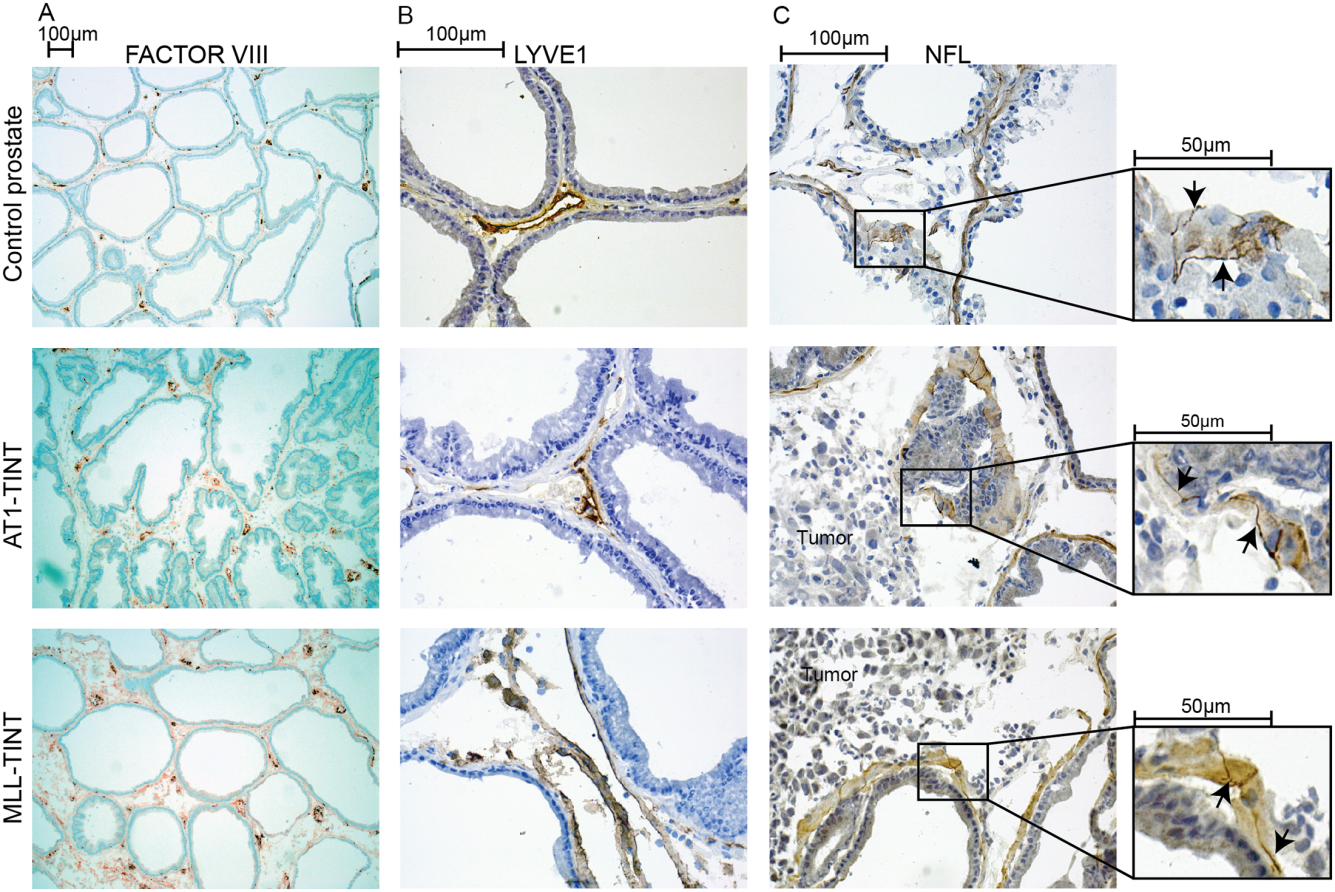
Immunostained tissue sections from control prostate, AT1-, and MLL-TINT. The densities of A) blood vessels (Factor VIII^+^, brown) and B) lymph vessels (LYVE1^+^, brown) and were higher in MLL-TINT than in AT1-TINT. C) Neurons (NFL^+^, brown) could be detected in the periglandular smooth muscle cell layer (arrows). Quantification showed that the density of NFL^+^ neurons was particularly decreased in AT1-TINT compared to the controls.

The blood vessel density (factor VIII^+^) was significantly higher in MLL-TINT (5.6 ± 2.2, n = 8) than in AT1-TINT (3.1 ± 0.69, p ≤0.05, n = 8) and treatment naïve controls (2.3 ± 0.46, p ≤ 0.05, n = 8) (Fig 8A). Lymph vessel density (Lyve1^+^) was significantly higher in MLL-TINT (1.2 ± 0.50, n = 8) compared to AT1-TINT (0.69 ± 0.15, p ≤ 0.05, n = 8) and treatment naïve controls (0.63 ± 0.21, p ≤ 0.05, n = 8) (Fig 8B). The volume density of CD3^+^ cells was higher in AT1-TINT (0.56 ± 0.15, n = 8) compared to MLL-TINT (0.33 ± 0.20, p ≤ 0.05, n = 8) and treatment naïve controls (0.20 ± 0.076, p ≤ 0.05, n = 8) (Fig 3A). The volume density of CD68^+^ cells was higher in MLL-TINT (0.88 ± 0.11, n = 7) compared to AT-TINT (0.62 ± 0.099, p ≤ 0.05, n = 8) and treatment naïve controls (0.20 ± 0.080, p ≤ 0.05, n = 7) (Fig 3B). The density of NFL^+^ nerve fibers was significantly reduced in both AT1-TINT (0.26 ± 0.052, n = 8) and in MLL-TINT (0.36 ± 0.13, n = 8) compared to treatment naïve controls (0.54 ± 0.18, n = 8, p ≤ 0.05) (Fig 8C).

Taken together and largely in line with the IPA analysis, this suggests that AT1-tumors were successful in attracting T-lymphocytes to the tumor-bearing organ whereas MLL-tumors attracted more macrophages and induced both blood- and lymphangiogenesis. Furthermore, nerves in the tumor-bearing organ could also be affected by the presence of a tumor.

### Gene expression profiles in AT1- and MLL-LNs

#### The most differentially expressed genes in AT1- and MLL-LNs

To examine differences induced in the regional LNs in the AT1- and MLL-models, we listed: 1) top 50 DEGs, 2) top 50 DEGs with a signal intensity value ≥ 500, and 3) top 25 most highly expressed DEGs (S6 Table).

Compared to controls, AT1- and MLL-associated LNs had numerous transcripts with similar changes in both tumor models, but unique responses were also seen for each tumor type (S6 Table). Several of the DEGs are known to be involved in immunosuppression, examples of such factors with a statistically significant higher expression in MLL- vs. AT1-LNs are *Il1r2*, *Ctla4*, *Pla2g7* but also *Lag3* (not in S6 Table but upregulated 1.3-fold) [76–79]. Some factors with a lower expression in MLL- vs. AT1- or control-LNs were: *Clec1b*—a factor needed for LN development and maintenance [80], and involved in maintaining the integrity of high endothelial venules [81], *C1ql2* –a regulator of inflammation and neurons [82], *Gzmm*–a factor that promotes killing of tumor cells [83], *Siglec1* (*Cd169*)–expressed by subcapsular sinus macrophages, important for capture and presentation of antigens and eliciting anti-tumor immune responses [84], *Gpr68* (*Ogr1*)–a factor needed for immune suppression [85], *Csf1* –a factor that promotes survival and differentiation of macrophages [86] including CD169^+^ macrophages [84], and its receptor *Csf1r* [87].

Genes related to tumor progression and metastasis were also differently expressed between MLL- and AT1-associated LNs. Genes with higher expression in MLL- vs. AT1-LNs whose gene products promote invasion and metastasis were for example, *Gpr55* [88], *Pla2g7* [78], and *Mir130b* [89]. Whereas genes associated with factors suppressing prostate tumor growth and metastasis like *Klf9*, and *Gpr68* [85, 90] were decreased in MLL- vs. AT1-LNs.

#### Gene ontology enrichment analysis of AT1- and MLL-associated LNs

The IPA core analysis of DEGs in LNs (FC ≥ 1.25, p ≤ 0.05) resulted in few significant differences between MLL- and AT1-LNs (the result of the entire downstream analysis, Diseases and functions annotations, are shown in S7 Table). In both models, the majority of the annotations were related to cell cycle and cell proliferation (Table 2), which suggests reactive LNs. Functional annotations related to angiogenesis, differentiation and connective tissue were also significantly altered (Table 2).

**Table 2:** GO enrichment analysis of regional LNs—Disease and function annotations.

Function annotation	Activation z-score			p-value			# associated genes (% associated genes predicted to increase/decrease/affect function)		
	MLL vs. control	AT1 vs. control	MLL vs. AT1	MLL vs. control	AT1 vs. control	MLL vs. AT1	MLL vs. control	AT1 vs. control	MLL vs. AT1
**Proliferation of cells**	3.0	1.0	-	3.93E-20	7.85E-12	-	883 (46/38/16)	538 (45/40/15)	-
**Migration of cells**	-3.5	-3.1	-	4.91E-12	3.22E-05	-	470 (36/50/14)	272 (36/52/12)	-
**Angiogenesis**	-4.9	-	-	9.65E-07	-	-	231 (24/53/23)	-	-
**Differentiation of cells**	-4.9	-	-	4.78E-06	-	-	507 (26/44/30)	-	-
**Chemotaxis of connective tissue cells**	-	-	-2.4	-	-	1.89E-03	-	-	6 (0/100/0)
**Activation of connective tissue cells**	-	-	-2.6	-	-	2.29E-02	-	-	9 (0/78/22)

IPA core analyses were performed for each comparison (MLL vs. control, AT1 vs. control, and MLL vs. AT1). DEGs with FC ≥ 1.25 and p ≤ 0.05 were included in the analyses. Selected disease and function annotations from the downstream effect analysis are listed in the table. MLL, n = 8; AT1, n = 8; control, n = 8. GO, Gene Ontology; IPA, Ingenuity Pathway Analysis; LN, Lymph Node; DEG, Differentially Expressed Gene; FC, Fold Change.

**Fig 9:**
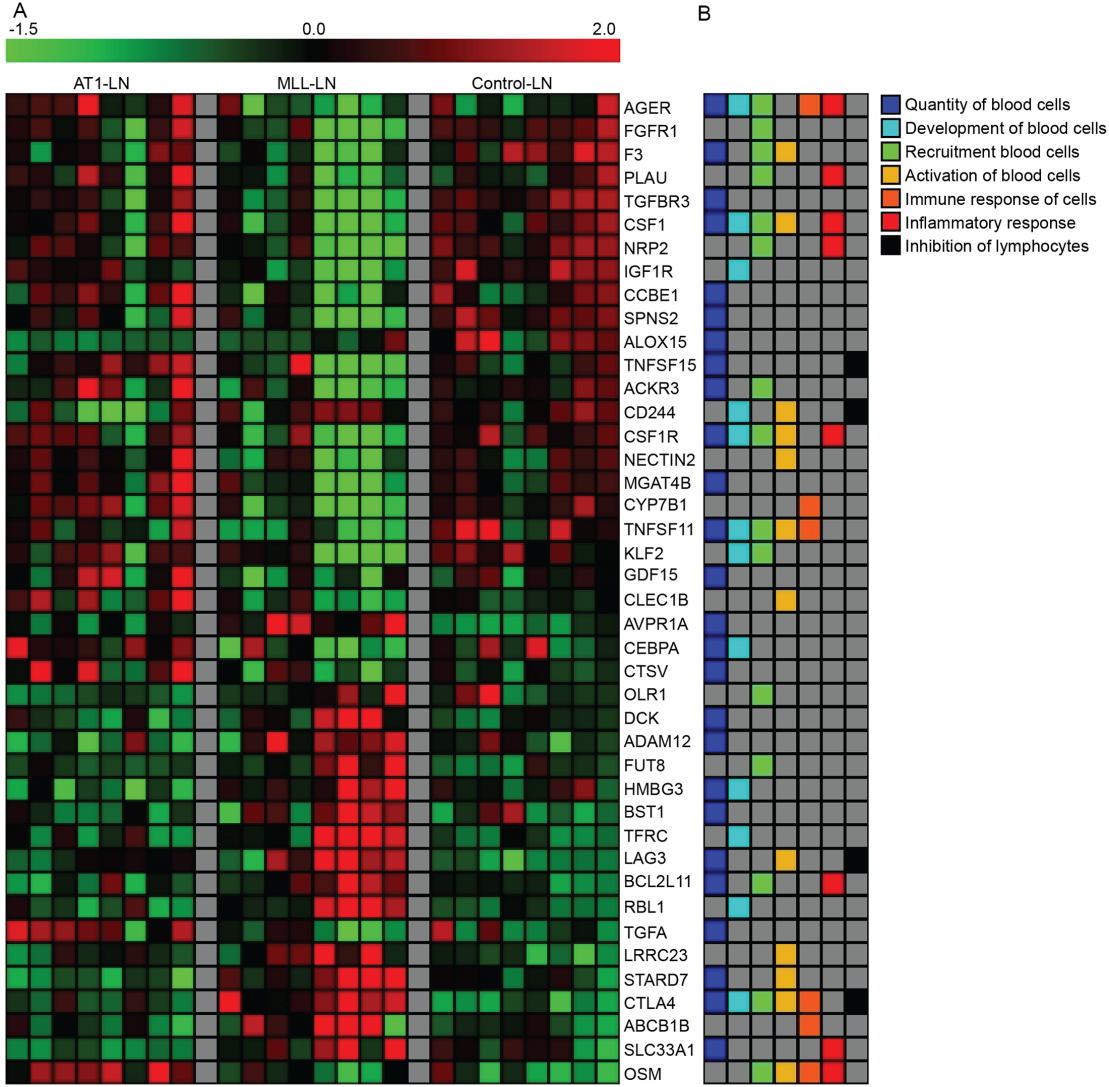
LN gene expression heatmap. A) Expression of DEGs associated with immune-related functions in LNs, as predicted by the IPA algorithm. All MLL- vs. AT1-LN DEGs in support of the prediction made by IPA are included. Gene expression signal intensity z-score is showed for each sample, black = mean signal intensity, red = number of standard deviations above the mean, green = number of standard deviations below the mean. B) The multicolored panel shows gene-function associations. MLL, n = 8; AT1, n = 8; control, n = 8. LN, Lymph Node; DEG, Differentially Expressed Gene; IPA, Ingenuity Pathway Analysis.

As the tumor-analysis suggested a more activated immune response in the AT1-tumors, we examined immune functions in the LNs more specifically. Examination of DEGs associated with functions within the categories *Hematological system development and function* and *Inflammatory response* resulted in a predicted decrease in for example quantity, differentiation, and activation of immune cells in MLL- vs. AT-LNs (data not shown). These predictions were made from a small number of genes (Fig 9) but still indicate differences in the adaptive immune response between MLL- and AT1-LNs. Some of these genes have already been mentioned among the most deregulated genes in the LNs, for example *Csf1*, *Csf1r*, *Clec1b*, *Lag3*, and *Ctla4* (see above). Other key factors associated with immune function, with a lower gene expression in MLL-LNs vs. AT1-LNs, were *Osm*–shown to induce formation of high endothelial venules and thereby affect recruitment of immune cells to the LN [91], and *Tnfs11* –regulates dendritic cell survival and LN formation [92] and enhance T cell response to tumor antigens [93]. Of the 42 DEGs in MLL- vs. AT1-LNs shown in Fig 9, only 3 (*adam12*, *cd244*, and *csf1r*) were correlated to tumor size (correlation coefficients 0.51, -0.53, and -0.53 respectively, p ≤ 0.05, data not shown) suggesting that tumor type influences LN gene expression more than tumor size.

From the IPA upstream analysis (S7 Table) we identified regulators that could be derived from the tumors given that they were differentially expressed in MLL- vs. AT1-tumors and were coding for proteins that could be secreted. These were CSF-1, TNF-alpha, TGF-beta1, IGF1, and CCL2 (all lower in MLL-tumors than in AT1-tumors and predicted to be inhibited upstream regulators in MLL- vs. AT1-LNs, data not shown). These factors were all directly associated with many of the functions shown in Fig 9, and can together with their downstream targets (for example CSF1R, TNFSF11, and OSM) possibly explain a decreased recruitment, development, differentiation and activation of immune cells in MLL-LNs compared to AT1-LNs. We could not find any obvious tumor-derived factors upstream of the upregulation of *Ctla4* and *Lag3* in the MLL-LNs. Neither could we find any differentially expressed miRNAs in the tumors that could be clearly linked to the gene expression differences between AT1- and MLL-LNs. The lists of possible tumor-derived factors that may influence gene expression in prostate TINT and regional LNs were largely non-overlapping.

#### Morphological differences and verification of selected ontology functions

The volume density (%) of CD3^+^ cells, Lyve1^+^ lymph vessels, factor VIII^+^ blood vessels and CD169^+^ sinus macrophages in LNs was not significantly different in vehicle-injected vs. treatment naive controls, although CD3 density tended to be increased by vehicle-injection (p = 0.06, data not shown).

**Fig 10:**
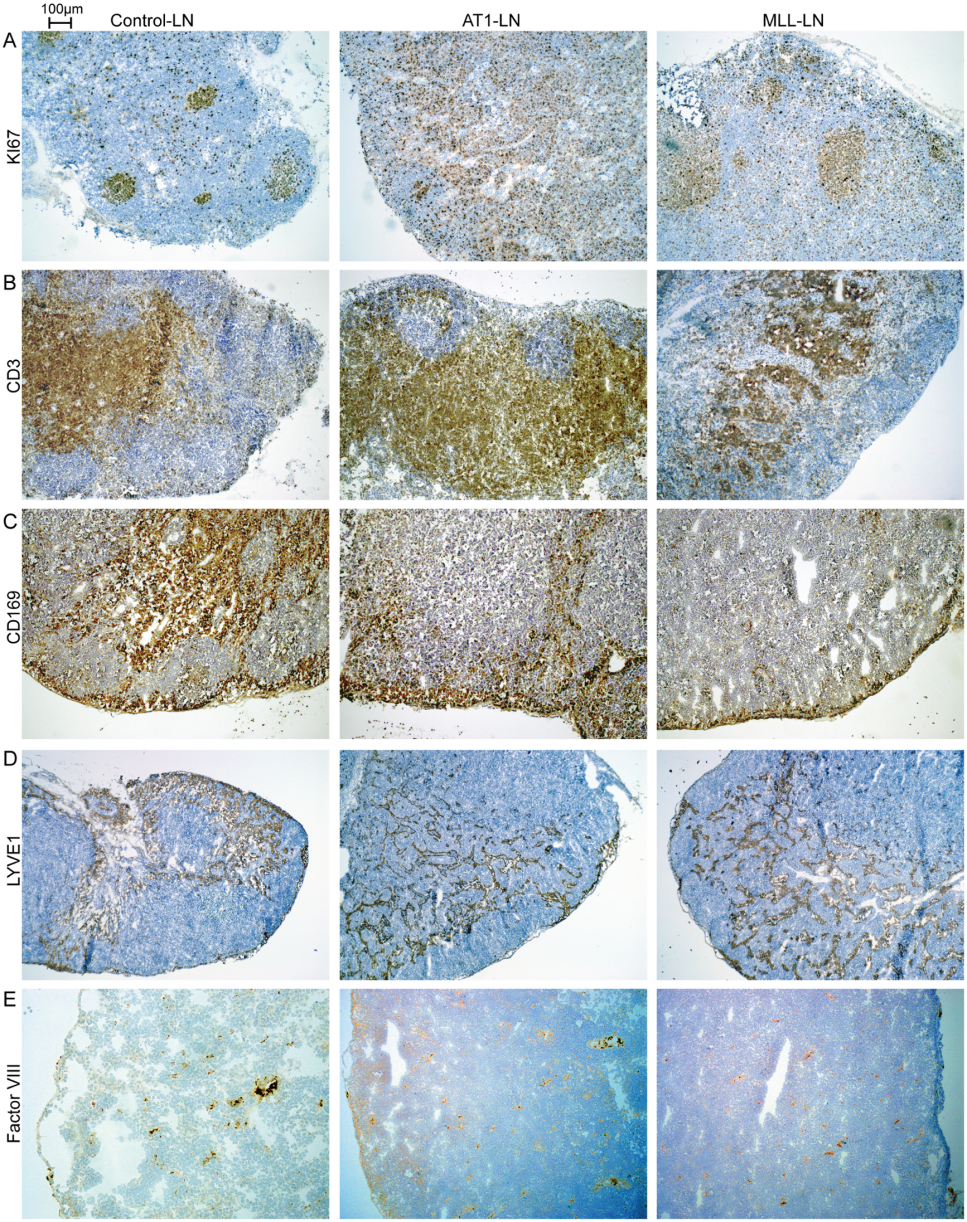
Immunostained tissue sections from control-, AT1-, and MLL-LNs. A) Proliferating cells (Ki67^+^, brown) were seen in lymph follicles (presumably B-lymphocytes) and in para-follicular areas (presumably T-lymphocytes) in control-LNs. In AT1-LNs, the para-follicular areas dominated, and contained most of the proliferating cells. In MLL-LNs, the para-follicular regions appeared to contain less proliferating cells than in AT1-LNs. B) CD3^+^ T-lymphocytes (brown) appeared more common in AT1-LNs compared to MLL-LNs, C) also sinus macrophages (CD169^+^, brown) appeared decreased in MLL-LNs. D) The density of LYVE1^+^ (brown) lymph vessels appeared similar in AT1- and MLL-LN. E) The density of Factor VIII^+^ (brown) blood vessels appeared reduced in MLL-LN.

The GO enrichment analysis in IPA suggested increased cell proliferation in both AT1- and MLL-LNs. In control-LNs, cell proliferation (measured as Ki67 labeling) was observed in lymph follicles and to some extent also in para-follicular regions. In AT1-LNs most of the proliferating cells were seen in expanded para-follicular regions, whereas proliferation in MLL-LNs was seen both in and around follicles (Fig 10A). The volume density of CD3^+^ lymphocytes was higher in AT1-LNs (0.74 ± 0.059, n = 7) compared to MLL-LNs (0.61 ± 0.097, p ≤ 0.05, n = 7) and control-LNs (0.54 ± 0.050, p ≤ 0.05, n = 6) (Fig 10B). Subcapsular sinus macrophages (CD169^+^) capture and present antigens in the LNs and thereby stimulate anti-tumor immune responses [84]. The volume density of CD169^+^ macrophages was lower in MLL-LNs (0.061 ± 0.017, n = 7) than in AT1-LNs (0.10 ± 0.025, p ≤ 0.05, n = 7) and than in treatment-naïve controls (0.11 ± 0.018, p ≤ 0.05, n = 6) (Fig 10C). This suggests that antigen presenting macrophages may decrease in the draining LNs of aggressive tumors.

The density of Lyve1^+^ vessel-like structures was similar in control-LNs (0.29 ± 0.10, n = 6), AT1-LNs (0.26 ± 0.11, n = 7) and MLL-LNs (0.29 ± 0.090, n = 7), but measurements of lymph vessel densities were difficult due the presence of other Lyve1^+^ cells, presumably macrophages (Fig 10D). The volume density of factor VIII^+^ blood vessels was similar in AT1-LNs (1.3 ± 0.50%, n = 6) and MLL-LNs (1.0 ± 0.42, n = 7), but for MLL-LNs this was significantly lower than the density of factor VIII in treatment naive controls (1.5 ± 0.42, n = 6, p ≤ 0.05) (Fig 10E).

## Discussion

This study was designed to describe adaptive extratumoral changes associated with subsequent metastatic disease, and to separate them from alterations induced by locally aggressive but poorly metastatic tumors. The locally aggressive but low metastatic AT1-tumor induced accumulation of T-lymphocytes in the tumor, in the tumor-bearing prostate and in regional LNs. In the benign parts of the prostate, cell proliferation, cell movement, cell survival, and growth of connective tissue and neurons appeared inhibited compared to that in animals with highly metastatic MLL-tumors. The host response at all these sites in the AT1-model therefore seemed to be an activated defense trying to restrict tumor growth and spread. MLL-tumors in contrast, induced an intratumoral innate immune response, vascular growth, and tumor-cell migration. In the benign prostate tissue next to MLL-tumors, we observed signs of increased inflammation, cell proliferation, cell survival, cell migration, and connective- and vascular tissue growth. Regional pre-metastatic LNs showed signs of decreased recruitment of antigen-presenting cells and possibly increased immunosuppression. This suggests that metastatic MLL-tumors are able to induce host responses in the tumor stroma, in the rest of the tumor-bearing organ, and in regional LNs that promotes tumor growth and spread.

We have previously shown that experimental prostate cancers influence the rest of the tumor-bearing organ, i.e. they induce extratumoral changes related to tumor aggressiveness [8]. We named such alterations as “TINT-changes”. TINT = tumor instructed (or induced, therefore also tumor indicating) normal tissue (see [6] for review). TINT-changes (adaptive changes in the previously normal epithelium and stroma) should be separated from signs of “field cancerization”. Field cancerization is defined as precancerous epithelial changes induced by a cancerogenic agent that has affected the entire organ and giving rise to both premalignant changes and induction of tumors [15, 94]. Field cancerization is consequently not present in our experimental model where cancer cells are injected into a normal organ. Importantly, and in support for the usefulness of the TINT-concept, we demonstrate that low- and high-metastatic tumors induce largely contrasting gene expression patterns in previously normal prostate tissue. This observation is in line with the general understanding that tumors may induce both inhibitory and stimulatory host responses. Our study shows that the tumor-bearing organ is an important, but somewhat neglected, site to search for tumor-induced host reactions. Normal tissues adjacent to tumors, and in particular the invasive zone, which is probably the primary battlefield between the tumor and the host, are probably sites where disease outcome could be determined. However, to avoid contamination we did not examine the gene expression in the invasive zone in the current study. Additional studies of changes in the TINT area closest to the tumor are therefore warranted. Further characterization of how tissues are “tinted”/reprogramed by different tumors can be used to identify novel prognostic markers, as well as novel therapeutic targets. More knowledge about the range and kinetics of such TINT responses are therefore important. As prostate needle biopsies only sample < 0.1% of the prostate volume (standard method to diagnose prostate cancer), they often fail to sample the most aggressive tumor present [6, 15]. Putative markers of disease aggressiveness in the benign prostate tissue—irrespective of whether they represent adaptive TINT-changes or precancerous lesions caused by field cancerization—could be useful in the clinic by indicating the presence of aggressive tumors elsewhere in the organ and thus aid in prostate cancer diagnosis and prognostication [6, 15].

In TINT surrounding the highly metastatic MLL-tumor we found multiple differently expressed genes encoding for proteins that are known to induce a tumor-promoting inflammation, a reactive stroma, and to promote invasion and metastasis (for example *Clu*, *Figf*, *Ccl2*, *Ctgf*, *Mmp15*, *Mmp7*, *Grhl3*, *Plau*, *Anxa2*, and *Bmp2*). The present study therefore suggests that metastatic tumors precondition major parts of the tumor-bearing prostate for subsequent tumor growth, invasion, and spread in ways resembling those described for the formation of a reactive tumor stroma and pre-metastatic niches. [1–3, 95]. The metastasis-promoting environment is probably formed already outside the tumor and later incorporated into the expanding tumor mass.

How TINT-changes are induced is largely unknown. Metastatic tumors probably produce and secrete factors, either as individual factors or packed in exosomes, reshaping the entire prostate. Several such upstream regulators and secreted factors were predicted in this study. However, the key factors and mechanisms involved remain to be established, as well as if the expression of these key factors is altered also at protein level. Exosomes from MLL-tumors are able to precondition (tint) normal prostate tissue for subsequent tumor growth [96] suggesting that this may be one mechanism. Among tumor-derived individual signals, our study suggests potential key roles for S100A9 and CXCL12 among others. These two factors have already been suggested to play important roles in establishing microenvironments favoring tumor growth and spread both in primary tumors and in pre-metastatic niches. Within TINT, increased activity of IFN-gamma, IL-1B, TGF-beta1, TNF, and prolactin may play central roles in forming an environment supporting tumor growth and spread. Fortunately, specific inhibitors for several of these potential upstream factors are available and should be used in future functional studies.

Are metastasis-associated TINT-changes detected in animals also present in prostate cancer patients and can they be used as diagnostic/prognostic markers? Some factors already identified in the rat model—such as *Hmox1*, *Lox*, and *Pdgfbr*, and accumulation of inflammatory cells such as macrophages, myeloid suppressor cells and mast cells—were increased in the benign parts of the prostate in cancer patients and the magnitudes of these changes were related to patient outcome [9, 11, 13, 65, 97]. In the present study, multiple additional TINT-markers related to metastatic disease were identified and their potential usefulness should be examined in patient samples. For example, *Ptx3*, upregulated in MLL-TINT only, was increased in histologically benign prostate epithelial cells adjacent to cancer in patients [98]. *Nkx3*.*1*, a key transcription factor regulating prostate epithelial differentiation [99], was particularly downregulated in MLL-TINT, but if a similar reduction is seen around aggressive human cancer needs to be examined. The role of nerves in TINT in relation to tumor aggressiveness should also be examined as increased nerve density in TINT is associated with aggressive disease in prostate cancer patients [100], but it was reduced in TINT surrounding both MLL- and AT1-tumors in our study.

Another underexplored site of novel diagnostic markers with the potential to predict metastatic disease in prostate cancer is tumor-free regional LNs. AT1-associated LNs were characterized by an increased density of CD3^+^ T-cells and maintained levels of antigen-presenting CD169^+^ cells probably securing accumulation of cytotoxic T-cells within and around the tumors. In patients with different cancer-types, increased T-cell numbers in regional tumor-free LNs is generally associated with a good prognosis [101]. In contrast, pre-metastatic MLL-LNs showed signs of decreased antigen presentation and an increased inhibition of immune responses. Individual factors responsible for this could for example be increased CTLA4 and LAG3 (blocking anti-tumor immune responses [79]), downregulated CSF-1, CSF-1R, TNFSF11 and CD169 (resulting in decreased antigen presentation [84]), and downregulated CLEC1B and OSM (resulting in decreased recruitment of immune cells via high endothelial venules [80, 81, 91]). As some of these factors can be specifically manipulated, and anti-CTLA4 therapies are already used to treat advanced prostate cancer patients [102, 103] future functional studies will be performed.

One of the main differences seen in the lymph node GO enrichment analysis was a predicted decrease in angiogenesis in MLL- vs. control-LNs. This is somewhat conflicting with current literature of angiogenesis in pre-metastatic LNs [23]. However, several factors involved in function or maintenance of high endothelial venules were downregulated in the MLL-LNs, for example *Osm* and *Clec1b*, suggesting that the predicted decrease in angiogenesis might instead reflect remodeling of high endothelial venules which have been reported to precede metastasis [23, 104, 105]. If this is the case also in our model needs to be further studied.

Further studies are needed to explore the kinetics of the suggested inhibition of anti-tumor immune responses in the pre-metastatic LNs and how responses to metastatic tumors can be differentiated from the responses to tumors that are more indolent or to other prostate diseases. In ongoing studies comparing the response over time in MLL- vs. AT1-LNs, we found factors that were selectively up- or downregulated in the MLL-LNs already 3 days after the tumor cell injection suggesting that a pre-metastatic response is detectable already when the tumors are very small (Strömvall et al 2017 unpublished). This raises the question whether only a few neoplastic cells are enough to signal to other organs or if the signal needs to be amplified by other cells in TINT, for example inflammatory cells. If present, signals amplified by non-malignant cells in TINT could potentially be useful as serum biomarkers for aggressive cancer, particularly as the TINT volume is usually considerably larger than the tumor volume (see [6] for discussion). In line with this, our study shows that many of the genes differentially expressed in prostate tumors were also changed in a similar way in TINT. In studies searching for prostate cancer biomarkers, tumor-adjacent non-malignant tissue is often used as a control. The current study therefore suggests that many potential biomarkers—i.e. markers that are changed in parallel in tumors and in the surrounding non-malignant tissue—could remain to be discovered. In line with our suggestion, gene expression of about 700 deregulated genes in the prostate tumor in patients were reported to be correlated (correlation coefficient > 0.7) to corresponding changes in the surrounding benign ipsi-lateral parts of the organ [106].

The question whether particularly aggressive human prostate tumors secrete factors that adapt regional LNs to future metastasis and if so at what time point, is largely unknown. However, increased vascular endothelial growth factor receptor 1 (VEGFR1) [29, 31] and Interleukin-30 [28] expressing cells in cancer-free LNs predicted subsequent metastatic disease after radical prostatectomy. Tumor-free LNs from prostate cancer patients contained more CD68^+^ and pSTAT-3^+^ macrophages than LNs from individuals without prostate cancer [24]. LNs containing prostate cancer metastases are often smaller than normal LNs and show signs of immunosuppression, and these responses may occur prior to arrival of metastatic cells [30]. These observations in patients and the current experimental findings suggest that the nature and kinetics of potential changes in pre-metastatic LNs need to be examined in more detail.

The gene expression pattern in a highly metastatic rat prostate tumor was different from that in a poorly metastatic variant. This is in line with numerous studies showing that tumor gene expression patterns in various tumor types, including the prostate, can be used to predict tumor aggressiveness [107]. Our GO enrichment analysis suggest that (as in other tumor types) this could partially be explained by different signals to, and responses within, the tumor microenvironment [5, 108]. Importantly our results also show differences in tumor-derived signals to, and responses within, the tumor-bearing prostate and regional LNs. Individual factors differentially expressed in high vs. low metastatic tumors could be linked to functional changes in the tumor-bearing organ and in regional LNs. However, the factors adapting the prostate for subsequent tumor growth and spread appeared different to those adapting the LNs.

In summary, the novel finding in this study is that metastasis can probably be predicted by adaptive pre-metastatic extratumoral changes in the rest of tumor-bearing organ and in regional LNs. Further studies are needed to examine the mechanisms behind as well as the range and kinetics of these host responses, and furthermore how they can be used to indirectly determine tumor aggressiveness and serve as novel therapeutic targets.

## Supporting information

S1 Dataset Whole genome cDNA microarray. Complete gene expression data set.
(XLSX)

S1 Fig Differentially expressed genes—Signal intensity and fold change distribution. A) FC distribution of DEGs in tumor, TINT and, LNs. B) Distribution of gene expression signal intensities of DEGs in tumor, TINT and LNs. C) Venn diagram of DEGs in tumor, TINT and LNs. MLL-tumor, n = 7; AT1-tumor, n = 8; MLL-TINT, n = 7; AT1-TINT, n = 6; MLL-LN, n = 8; AT1-LN, n = 8; control-prostate, n = 8; control-LN, n = 8. FC, Fold Change; DEG, Differentially Expressed Gene; TINT, Tumor Instructed Normal Tissue; LN, Lymph Node. Comments to S1 Fig: When compared to controls, AT1- and MLL-tumors had almost the same number of DEGs, but MLL-tumors had more downregulated genes with a large FC. Most of the DEGs seen in each tumor type were shared, however, due to differences in magnitudes about half of them differ between MLL and AT1, and both tumor types also have a unique set of DEGs. In prostate TINT, most of the DEGs seen in each tumor-model were unique. AT1-TINT had almost twice as many DEGs as MLL-TINT. The majority of DEGs (75%) in AT1-TINT were downregulated, while the majority (69%) of DEGs in MLL-TINT were upregulated. A substantial part (27%) of the DEGs in MLL- vs. AT1-TINT comparison were contraregulated with small FC (being non-significant in each model alone) and most of the remaining part of DEGs were changes exclusive for AT1-TINT. The gene expression in MLL-LNs was more different to control-LNs than that in AT1-LNs. Still, the list of DEGs obtained when comparing MLL- to AT1-LNs was limited. Half of it was composed of genes identified in MLL-LNs alone, and most of the rest were contraregulated genes with small FC (as in TINT, being non-significant in each model alone).
(TIF)

S2 Fig GO enrichment analysis of tumors—Immune-related disease and function annotations. IPA core analyses were performed for each comparison (MLL vs. control, AT1 vs. control, and MLL vs. AT1). DEGs with FC ≥ 1.5 and p ≤ 0.05 were included in the analyses. Significant (p ≤ 0.05, z-score > 2 (absolute value)) immune-related function annotations are shown. The heatmap illustrates the predicted activation z-scores, blue = negative score, decreased activity, and orange = positive score, increased activity. MLL, n = 7; AT1, n = 8; control, n = 8. GO, Gene Ontology; IPA, Ingenuity Pathway Analysis; DEG, Differentially Expressed Gene; FC, Fold Change. Reprinted from IPA under a CC BY license, with permission from Qiagen, original copyright 2016.
(TIF)

S1 Table **A) Tumor—Top 50 DEGs. B) Tumor—Top 50 DEGs with a signal intensity value ≥ 500. C) Tumor—Top 25 most highly expressed DEGs**. DEG, Differentially expressed gene (FC ≥ 1.25, p ≤ 0.05); FC, Fold change.
(DOCX)

S2 Table GO enrichment analysis of tumors—Disease and function annotations. IPA core analyses were performed for each comparison (MLL vs. control, AT1 vs. control, and MLL vs. AT1). DEGs with FC ≥ 1.5 and p ≤ 0.05 were included in the analyses. Selected disease and function annotations from the downstream effect analysis are listed in the table. MLL, n = 7; AT1, n = 8; control, n = 8. GO, Gene ontology; IPA, Ingenuity Pathway Analysis; DEG, Differentially Expressed Gene; FC, Fold Change.
(DOCX)

S3 Table GO enrichment analysis of tumors. IPA core analyses were performed for each comparison (MLL vs. control, AT1 vs. control, and MLL vs. AT1). DEGs with FC ≥ 1.5 and p ≤ 0.05 were included in the analyses. The entire result of the sections Diseases and Functions, and Upstream Regulators are shown. MLL, n = 7; AT1, n = 8; control, n = 8. GO, Gene ontology; IPA, Ingenuity Pathway Analysis; DEG, Differentially Expressed Gene; FC, Fold Change.
(XLSX)

S4 Table **A) TINT—Top 50 DEGs. B) TINT—Top 50 DEGs with a signal intensity value ≥ 500. C) TINT—Top 25 most highly expressed DEGs**. DEG, Differentially expressed gene (FC ≥ 1.25, p ≤ 0.05); FC, Fold change; TINT, Tumor Instructed Normal Tissue.
(DOCX)

S5 Table GO enrichment analysis of prostate TINT. IPA core analyses were performed for each comparison (MLL vs. control, AT1 vs. control, and MLL vs. AT1). DEGs with FC ≥ 1.25 and p ≤ 0.05 were included in the analyses. The entire result of the sections Diseases and Functions, and Upstream Regulators are shown. MLL, n = 7; AT1, n = 6; control, n = 8. GO, Gene Ontology; TINT, Tumor Instructed Normal Tissue; IPA, Ingenuity Pathway Analysis; DEG, Differentially Expressed Gene; FC, Fold Change.
(XLSX)

S6 Table **A) LN—Top 50 DEGs. B) LN—Top 50 DEGs with a signal intensity value ≥ 500. C) LN—Top 25 most highly expressed DEGs**. DEG, Differentially expressed gene (FC ≥ 1.25, p ≤ 0.05); FC, Fold change; LN, Lymph Node.
(DOCX)

S7 Table GO enrichment analysis of regional LNs. IPA core analyses were performed for each comparison (MLL vs. control, AT1 vs. control, and MLL vs. AT1). DEGs with FC ≥ 1.25 and p ≤ 0.05 were included in the analyses. The entire result of the sections Diseases and Functions, and Upstream Regulators are shown. MLL, n = 8; AT1, n = 8; control, n = 8. GO, Gene Ontology; IPA, Ingenuity Pathway Analysis; LN, Lymph Node; DEG, Differentially Expressed Gene; FC, Fold Change.
(XLSX)

S1 Copyright Permission Permission to publish copyrighted figures under the Creative Commons Attribution License (CCAL) CC BY 4.0. (PDF)
