# Supplementary figures and images for: Aggressive rat prostate tumors reprogram the benign parts of the prostate and regional lymph nodes prior to metastasis
Source: PLoS One. 2017 May 4;12(5):e0176679. doi: 10.1371/journal.pone.0176679 (PMC5417597; doi:10.1371/journal.pone.0176679)

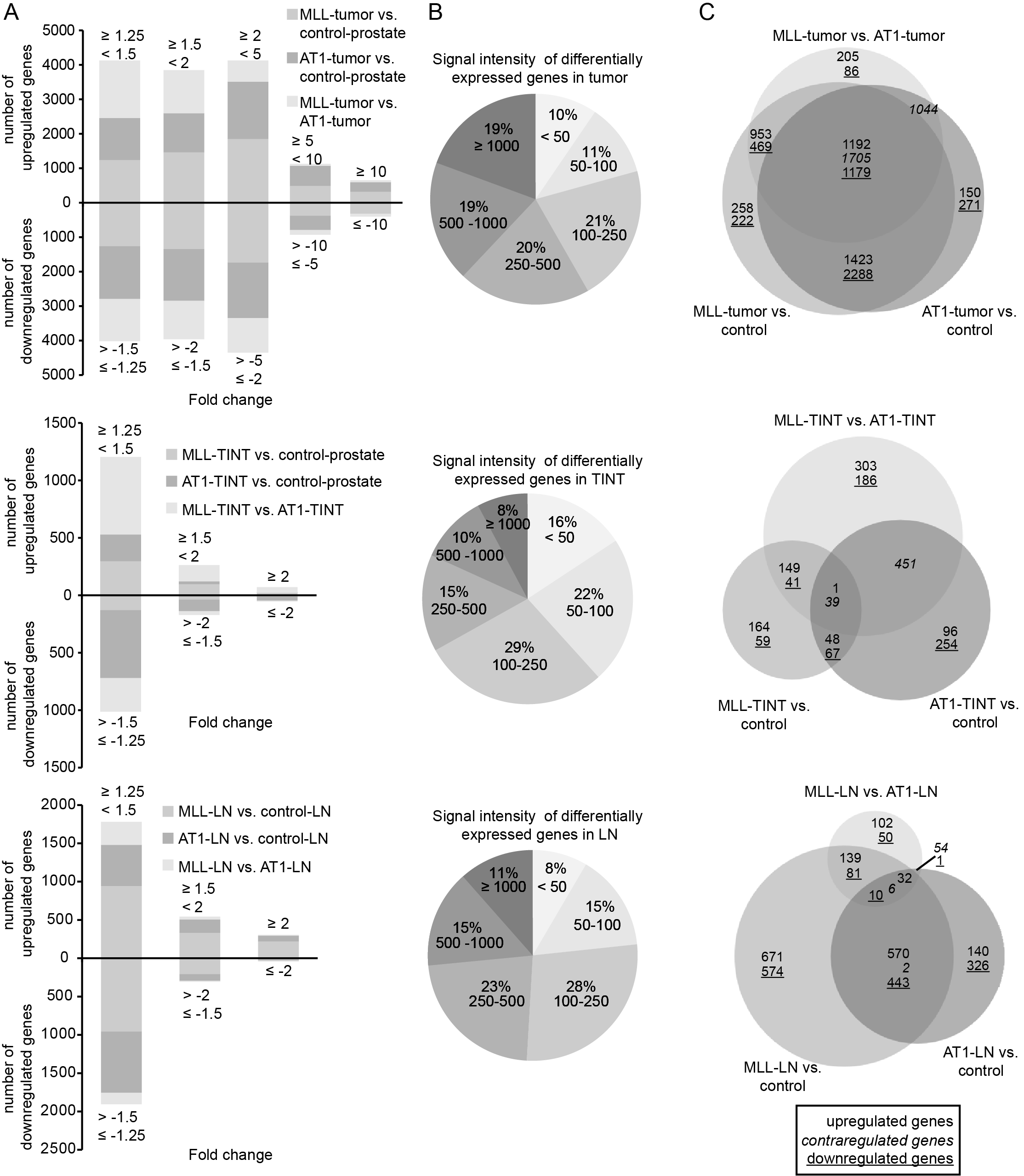

Supplement: S1 Fig — A) FC distribution of DEGs in tumor, TINT and, LNs. B) Distribution of gene expression signal intensities of DEGs in tumor, TINT and LNs. C) Venn diagram of DEGs in tumor, TINT and LNs. MLL-tumor, n = 7; AT1-tumor, n = 8; MLL-TINT, n = 7; AT1-TINT, n = 6; MLL-LN, n = 8; AT1-LN, n = 8; control-prostate, n = 8; control-LN, n = 8. FC, Fold Change; DEG, Differentially Expressed Gene; TINT, Tumor Instructed Normal Tissue; LN, Lymph Node. Comments to S1 Fig: When compared to controls, AT1- and MLL-tumors had almost the same number of DEGs, but MLL-tumors had more downregulated genes with a large FC. Most of the DEGs seen in each tumor type were shared, however, due to differences in magnitudes about half of them differ between MLL and AT1, and both tumor types also have a unique set of DEGs. In prostate TINT, most of the DEGs seen in each tumor-model were unique. AT1-TINT had almost twice as many DEGs as MLL-TINT. The majority of DEGs (75%) in AT1-TINT were downregulated, while the majority (69%) of DEGs in MLL-TINT were upregulated. A substantial part (27%) of the DEGs in MLL- vs. AT1-TINT comparison were contraregulated with small FC (being non-significant in each model alone) and most of the remaining part of DEGs were changes exclusive for AT1-TINT. The gene expression in MLL-LNs was more different to control-LNs than that in AT1-LNs. Still, the list of DEGs obtained when comparing MLL- to AT1-LNs was limited. Half of it was composed of genes identified in MLL-LNs alone, and most of the rest were contraregulated genes with small FC (as in TINT, being non-significant in each model alone). (TIF) [file pone.0176679.s002.tif]

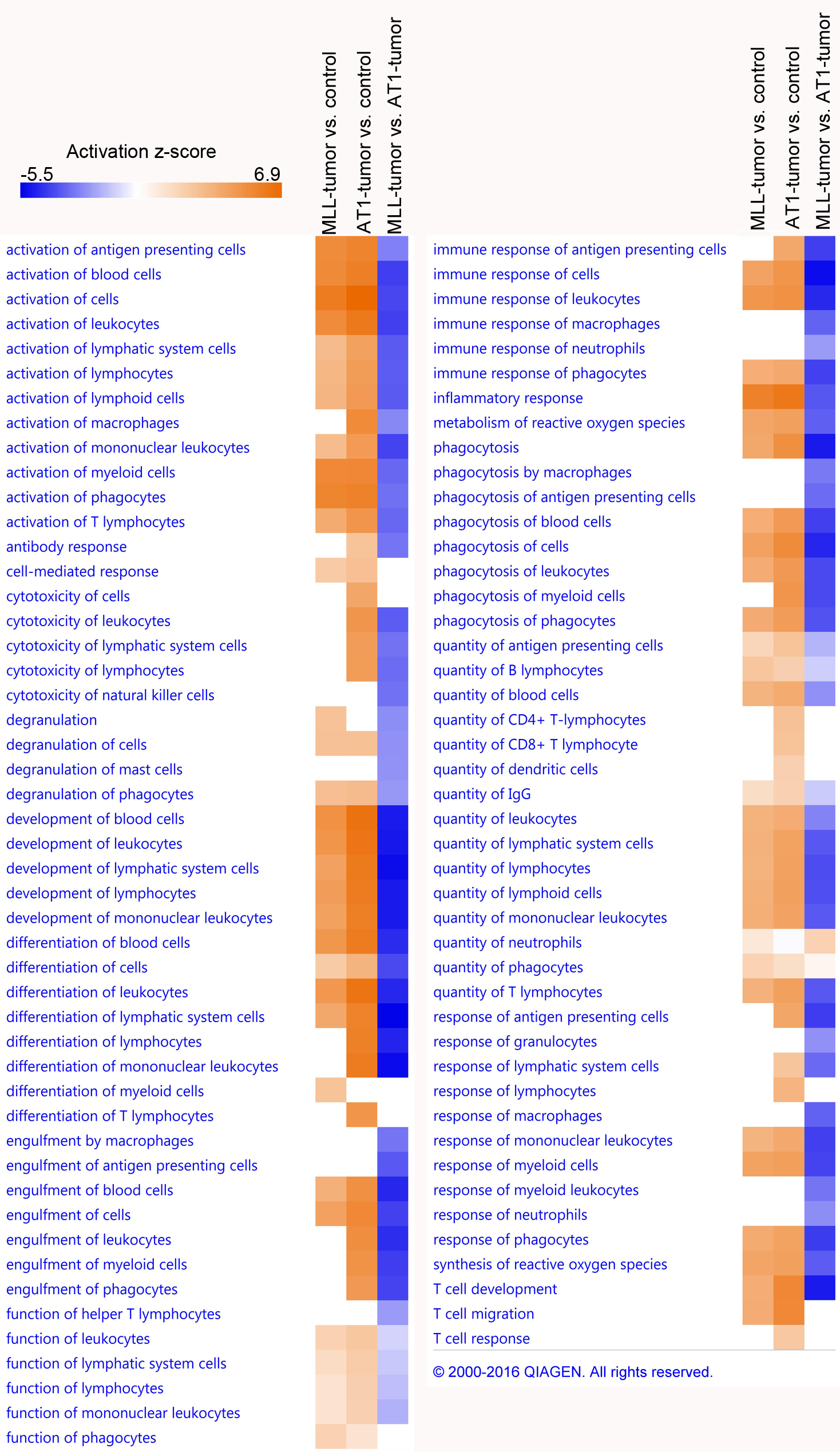

Supplement: S2 Fig — IPA core analyses were performed for each comparison (MLL vs. control, AT1 vs. control, and MLL vs. AT1). DEGs with FC ≥ 1.5 and p ≤ 0.05 were included in the analyses. Significant (p ≤ 0.05, z-score > 2 (absolute value)) immune-related function annotations are shown. The heatmap illustrates the predicted activation z-scores, blue = negative score, decreased activity, and orange = positive score, increased activity. MLL, n = 7; AT1, n = 8; control, n = 8. GO, Gene Ontology; IPA, Ingenuity Pathway Analysis; DEG, Differentially Expressed Gene; FC, Fold Change. Reprinted from IPA under a CC BY license, with permission from Qiagen, original copyright 2016. (TIF) [file pone.0176679.s003.tif]
