# Supplementary material for: Aggressive rat prostate tumors reprogram the benign parts of the prostate and regional lymph nodes prior to metastasis
Source: PLoS One. 2017 May 4;12(5):e0176679. doi: 10.1371/journal.pone.0176679 (PMC5417597; doi:10.1371/journal.pone.0176679)
Supplement: S1 Table — A) Tumor—Top 50 DEGs. B) Tumor—Top 50 DEGs with a signal intensity value ≥ 500. C) Tumor—Top 25 most highly expressed DEGs. DEG, Differentially expressed gene (FC ≥ 1.25, p ≤ 0.05); FC, Fold change. (DOCX) [file pone.0176679.s004.docx]

**S1 Table. A) Tumor - Top 50 DEGs**

| **MLL-tumor vs. control-prostate** | | **AT1-tumor vs. control-prostate** | | **MLL-tumor vs. AT1-tumor** | |
| --- | --- | --- | --- | --- | --- |
| **Gene** **symbol** | **FC** | **Gene symbol** | **FC** | **Gene symbol** | **FC** |
| **Upregulated genes** | | | | | |
| LOC290595 | 167.0 | LOC100363492 | 217.0 | LOC679119 | 117.0 |
| Cyp3a9 | 133.0 | LOC290595 | 177.0 | Olr1684 | 96.9 |
| Pmp2 | 103.0 | Serpinb2 | 136.0 | Pmp2 | 95.5 |
| LOC679119 | 99.2 | Nppb | 131.0 | Th | 49.4 |
| Olr1684 | 98.2 | Mmp3 | 126.0 | RGD1563703 | 43.4 |
| Kbtbd10 | 97.4 | Matn3 | 119.0 | Fam5c | 42.4 |
| Serpinb2 | 97.1 | Clec2dl1 | 73.1 | Cxcl6 | 40.5 |
| Lrrn3 | 83.7 | Crabp2 | 66.7 | Armcx2 | 37.9 |
| Gzmb | 76.9 | LOC497860 | 59.0 | Cdh6 | 37.0 |
| Crabp1 | 67.9 | Thbs4 | 52.7 | Pcbd1 | 36.8 |
| Ptprn | 67.6 | S100a4 | 46.5 | Diras2 | 32.5 |
| Cda | 67.1 | Rpl10l | 44.7 | Pi15 | 31.1 |
| Clec2dl1 | 65.6 | P4ha3 | 39.8 | Fabp4 | 29.6 |
| Nav3 | 59.4 | Cdx2 | 39.3 | Eya2 | 29.0 |
| Th | 59.3 | Cyp3a9 | 39.2 | Kbtbd10 | 28.5 |
| Car9 | 58.0 | Mmp10 | 39.0 | Ptprn | 26.6 |
| Grip1 | 57.8 | Slc16a3 | 38.1 | Chrdl1 | 26.3 |
| Fosl1 | 56.9 | Slurp1 | 37.4 | Lsamp | 25.8 |
| Steap1 | 51.7 | RGD1307595 | 37.1 | Sgsm1 | 24.4 |
| S100a4 | 49.9 | Lilrb4 | 36.2 | Epgn | 23.5 |
| Aldh3a1 | 48.2 | F2rl2 | 36.0 | Sox2 | 23.1 |
| Mgst2 | 46.0 | Fcnb | 35.7 | Cxcl2 | 22.4 |
| Armcx2 | 45.0 | Akr1b8 | 33.4 | Rab9b | 21.1 |
| Fam5c | 44.7 | Olr1014 | 32.4 | Bmp5 | 21.0 |
| Mybl2 | 41.4 | Nkain1 | 32.1 | Cda | 18.2 |
| **Downregulated genes** | | | | | |
| Srd5a2 | -40.9 | Nefl | -15.3 | LOC100363492 | -212.0 |
| Klk1b21 | -39.9 | Nkx3-1 | -14.0 | Matn3 | -118.0 |
| Stx19 | -38.4 | Srd5a2 | -13.2 | Thbs4 | -73.3 |
| Olr660 | -37.1 | Olr660 | -12.3 | LOC497860 | -62.0 |
| Nxph1 | -34.1 | MGC109340 | -12.1 | Dsc3 | -61.9 |
| Ugt8 | -33.7 | Stx19 | -11.5 | Rpl10l | -60.5 |
| Sytl5 | -31.7 | Esr2 | -10.4 | RGD1307595 | -38.2 |
| Nefl | -31.3 | Gstm6l | -10.4 | Nlrp4 | -36.4 |
| Cyp7b1 | -31.1 | Lgr5 | -10.2 | Fbln2 | -33.8 |
| Pcdh10 | -30.4 | Gstm7 | -9.9 | Khdrbs3 | -32.4 |
| Esr2 | -30.1 | Slc5a8 | -9.8 | Mmp10 | -31.6 |
| Slc7a4 | -30.1 | Frem2 | -9.8 | MGC114427 | -28.6 |
| Zfp185 | -29.2 | Acrbp | -9.7 | Ptprv | -24.9 |
| LOC685203 | -29.2 | Prom2 | -9.7 | Slurp1 | -24.4 |
| Cwh43 | -28.6 | Fmo2 | -9.6 | Gabra1 | -21.2 |
| Cd99l2 | -28.5 | Sptlc3 | -9.4 | Cnih2 | -20.0 |
| Eaf2 | -28.2 | Ldoc1 | -9.3 | Lrrn4cl | -19.8 |
| Pik3c2g | -27.9 | Ces3 | -9.3 | Mmp3 | -19.1 |
| Elovl7 | -27.9 | Cbs | -9.3 | Ncam1 | -18.0 |
| Timp4 | -27.7 | Dhtkd1 | -9.3 | Mageb1 | -17.9 |
| Prom2 | -27.6 | Sult1d1 | -9.3 | Sv2b | -17.0 |
| Sptlc3 | -27.5 | Abat | -9.3 | Nxf7 | -16.7 |
| Snca | -26.9 | Pik3c2g | -9.2 | Anxa8 | -16.5 |
| Ldoc1 | -26.3 | Snca | -9.1 | RGD1562449 | -16.5 |
| Tmem54 | -25.6 | Nxph1 | -9.1 | Ard1b | -16.0 |

DEG, Differentially expressed gene (FC ≥ 1.25, p ≤ 0.05); FC, Fold Change

**S1 Table. B) Tumor – top 50 DEGs with a signal intensity value ≥ 500**

| **MLL-tumor vs. control-prostate** | | **AT1-tumor vs. control-prostate** | | **MLL-tumor vs. AT1-tumor** | |
| --- | --- | --- | --- | --- | --- |
| **Gene** **symbol** | **FC** | **Gene symbol** | **FC** | **Gene symbol** | **FC** |
| **Upregulated genes** | | | | | |
| LOC290595 | 167.0 | LOC100363492 | 217.0 | LOC679119 | 117.0 |
| Cyp3a9 | 133.0 | LOC290595 | 177.0 | Olr1684 | 96.9 |
| Pmp2 | 103.0 | Serpinb2 | 136.0 | Pmp2 | 95.5 |
| LOC679119 | 99.2 | Nppb | 131.0 | Th | 49.4 |
| Olr1684 | 98.2 | Mmp3 | 126.0 | RGD1563703 | 43.4 |
| Kbtbd10 | 97.4 | Matn3 | 119.0 | Fam5c | 42.4 |
| Serpinb2 | 97.1 | Clec2dl1 | 73.1 | Cxcl6 | 40.5 |
| Lrrn3 | 83.7 | Crabp2 | 66.7 | Armcx2 | 37.9 |
| Gzmb | 76.9 | LOC497860 | 59.0 | Cdh6 | 37.0 |
| Crabp1 | 67.9 | Thbs4 | 52.7 | Pcbd1 | 36.8 |
| Ptprn | 67.6 | S100a4 | 46.5 | Diras2 | 32.5 |
| Cda | 67.1 | Rpl10l | 44.7 | Fabp4 | 29.6 |
| Clec2dl1 | 65.6 | P4ha3 | 39.8 | Eya2 | 29.0 |
| Nav3 | 59.4 | Slc16a3 | 38.1 | Kbtbd10 | 28.5 |
| Th | 59.3 | Slurp1 | 37.4 | Ptprn | 26.6 |
| Car9 | 58.0 | RGD1307595 | 37.1 | Chrdl1 | 26.3 |
| Grip1 | 57.8 | Lilrb4 | 36.2 | Lsamp | 25.8 |
| Fosl1 | 56.9 | F2rl2 | 36.0 | Sgsm1 | 24.4 |
| Steap1 | 51.7 | Fcnb | 35.7 | Sox2 | 23.1 |
| S100a4 | 49.9 | Akr1b8 | 33.4 | Rab9b | 21.1 |
| Aldh3a1 | 48.2 | Nkain1 | 32.1 | Cda | 18.2 |
| Mgst2 | 46.0 | RGD1560455 | 31.8 | Clic5 | 18.1 |
| Armcx2 | 45.0 | Tmem119 | 31.3 | Ifit1 | 16.8 |
| Fam5c | 44.7 | Dsc3 | 30.0 | Esm1 | 16.0 |
| Mybl2 | 41.4 | Adam12 | 29.8 | Mecom | 14.9 |
| **Downregulated genes** | | | | | |
| Srd5a2 | -40.9 | Srd5a2 | -13.2 | LOC100363492 | -212.0 |
| Klk1b21 | -39.9 | Olr660 | -12.3 | Matn3 | -118.0 |
| Stx19 | -38.4 | MGC109340 | -12.1 | Thbs4 | -73.3 |
| Olr660 | -37.1 | Stx19 | -11.5 | LOC497860 | -62.0 |
| Nxph1 | -34.1 | Esr2 | -10.4 | Dsc3 | -61.9 |
| Ugt8 | -33.7 | Gstm6l | -10.4 | Rpl10l | -60.5 |
| Sytl5 | -31.7 | Gstm7 | -9.9 | RGD1307595 | -38.2 |
| Cyp7b1 | -31.1 | Slc5a8 | -9.8 | Fbln2 | -33.8 |
| Pcdh10 | -30.4 | Frem2 | -9.8 | Khdrbs3 | -32.4 |
| Esr2 | -30.1 | Acrbp | -9.7 | Slurp1 | -24.4 |
| Slc7a4 | -30.1 | Prom2 | -9.7 | Cnih2 | -20.0 |
| Zfp185 | -29.2 | Ldoc1 | -9.3 | Lrrn4cl | -19.8 |
| LOC685203 | -29.2 | Abat | -9.3 | Mmp3 | -19.1 |
| Cwh43 | -28.6 | Pik3c2g | -9.2 | Ncam1 | -18.0 |
| Cd99l2 | -28.5 | Snca | -9.1 | Mmp2 | -15.0 |
| Eaf2 | -28.2 | Nxph1 | -9.1 | Mfap3l | -14.6 |
| Pik3c2g | -27.9 | Slc7a4 | -9.0 | Tshz3 | -14.6 |
| Elovl7 | -27.9 | Timp4 | -8.9 | Aebp1 | -14.5 |
| Timp4 | -27.7 | Prkaa2 | -8.7 | Cercam | -13.1 |
| Prom2 | -27.6 | LOC683719 | -8.7 | Gpx3 | -12.8 |
| Snca | -26.9 | Cd99l2 | -8.7 | Gnai1 | -12.6 |
| Ldoc1 | -26.3 | Svs4 | -8.5 | Ccrl1 | -12.3 |
| Tmem54 | -25.6 | Frmpd4 | -8.5 | Cdh11 | -12.1 |
| Mal2 | -25.6 | Esrp2 | -8.5 | Htra3 | -11.3 |
| Rdh2 | -25.3 | Zfp185 | -8.4 | Rcn3 | -11.2 |

DEG, Differentially expressed gene (FC ≥ 1.25, p ≤ 0.05); FC, Fold Change

**S1 Table. C) Tumor – top 25 most highly expressed DEGs**

| **MLL-tumor vs. control-prostate** | | | | **AT1-tumor vs. control-prostate** | | | | **MLL-tumor vs. AT1-tumor** | | | |
| --- | --- | --- | --- | --- | --- | --- | --- | --- | --- | --- | --- |
| **Gene** | **Intensity MLL** | **Intensity control** | **FC** | **Gene** | **Intensity AT1** | **Intensity control** | **FC** | **Gene** | **Intensity MLL** | **Intensity AT1** | **FC** |
| Klks3 | 2410 | 11407 | -4.73 | Klks3 | 5142 | 11407 | -2.22 | Cd74 | 6284 | 7985 | -1.27 |
| Gapdh | 10985 | 6746 | 1.63 | Rps20 | 10853 | 8455 | 1.28 | Mt2A | 7720 | 2868 | 2.69 |
| Rps20 | 10694 | 8455 | 1.26 | Hba-a2 | 6476 | 10001 | -1.54 | Sparc | 3987 | 7631 | -1.91 |
| Hba-a2 | 6594 | 10001 | -1.52 | LOC296235 | 5932 | 9611 | -1.62 | Lyz2 | 4380 | 6934 | -1.58 |
| LOC296235 | 3588 | 9611 | -2.68 | Psbpc1 | 6699 | 9334 | -1.39 | Hist2h3c2 | 6742 | 5320 | 1.27 |
| Actg1 | 9487 | 5998 | 1.58 | Sbp | 5282 | 9155 | -1.73 | Psbpc1 | 4024 | 6699 | -1.66 |
| Psbpc1 | 4024 | 9334 | -2.32 | Prp2l1 | 5211 | 8909 | -1.71 | Ywhaq | 6579 | 5097 | 1.29 |
| Sbp | 2930 | 9155 | -3.12 | LOC100362601 | 3861 | 8615 | -2.23 | Bgn | 1152 | 6315 | -5.48 |
| Ldha | 9126 | 2124 | 4.30 | Spcs1 | 4756 | 8225 | -1.73 | Ctsb | 4189 | 6168 | -1.47 |
| Prp2l1 | 2404 | 8909 | -3.71 | B2m | 7862 | 5074 | 1.55 | Prelid1 | 5912 | 4297 | 1.38 |
| LOC100362601 | 1599 | 8615 | -5.39 | Hspa5 | 5326 | 7823 | -1.47 | Slc25a5 | 5863 | 4661 | 1.26 |
| Hist1h4b | 8421 | 2346 | 3.59 | Psbpc2 | 5365 | 7745 | -1.44 | RT1-Da | 3566 | 5825 | -1.63 |
| Spcs1 | 4888 | 8225 | -1.68 | Andpro | 4785 | 7674 | -1.60 | Psap | 4368 | 5823 | -1.33 |
| Hspa5 | 5368 | 7823 | -1.46 | Rmrp | 7296 | 5712 | 1.28 | Nap1l1 | 5724 | 3166 | 1.81 |
| Psbpc2 | 3234 | 7745 | -2.39 | Perp | 2966 | 6969 | -2.35 | Eif4b | 5629 | 4464 | 1.26 |
| Mt2A | 7720 | 2327 | 3.32 | Mme | 1754 | 6940 | -3.96 | Cdk4 | 5592 | 4246 | 1.32 |
| Andpro | 2490 | 7674 | -3.08 | Calr | 4965 | 6871 | -1.38 | Hist2h4 | 5524 | 4240 | 1.30 |
| Hist2h3c2 | 7443 | 1861 | 4.00 | Gabarap | 5323 | 6815 | -1.28 | Cdk2ap1 | 5467 | 3390 | 1.61 |
| Vim | 7416 | 2547 | 2.91 | Scgb2a2 | 5343 | 6788 | -1.27 | Psbpc2 | 3234 | 5365 | -1.66 |
| Hist1h2bc | 7378 | 2707 | 2.73 | Cmah | 1780 | 6697 | -3.76 | Scgb2a2 | 4096 | 5343 | -1.30 |
| Pdia3 | 5413 | 7256 | -1.34 | Dstn | 4929 | 6627 | -1.34 | Gabarap | 3842 | 5323 | -1.39 |
| Cav1 | 7230 | 2022 | 3.58 | Scgb2a1 | 5034 | 6389 | -1.27 | Hist1h2ail | 5308 | 4149 | 1.28 |
| Ftl | 7207 | 4786 | 1.51 | Cd24 | 1673 | 6332 | -3.79 | Sbp | 2930 | 5282 | -1.80 |
| Tuba1c | 7182 | 4347 | 1.65 | Ptplb | 2164 | 6326 | -2.92 | Lman2 | 5271 | 4019 | 1.31 |
| Perp | 1309 | 6969 | -5.32 | Rps2 | 6287 | 4821 | 1.30 | Hdgf | 5224 | 3374 | 1.55 |

DEG, Differentially expressed gene (FC ≥ 1.25, p ≤ 0.05); FC, Fold change
