# Supplementary material for: Aggressive rat prostate tumors reprogram the benign parts of the prostate and regional lymph nodes prior to metastasis
Source: PLoS One. 2017 May 4;12(5):e0176679. doi: 10.1371/journal.pone.0176679 (PMC5417597; doi:10.1371/journal.pone.0176679)
Supplement: S2 Table — IPA core analyses were performed for each comparison (MLL vs. control, AT1 vs. control, and MLL vs. AT1). DEGs with FC ≥ 1.5 and p ≤ 0.05 were included in the analyses. Selected disease and function annotations from the downstream effect analysis are listed in the table. MLL, n = 7; AT1, n = 8; control, n = 8. GO, Gene ontology; IPA, Ingenuity Pathway Analysis; DEG, Differentially Expressed Gene; FC, Fold Change. (DOCX) [file pone.0176679.s005.docx]

**S2 Table. GO enrichment analysis of tumors - Disease and function annotations.**

| **Function annotation** | **Activation z-score** | | | **p-value** | | | **# associated genes (% associated genes predicted to increase/decrease/affect function)** | | |
| --- | --- | --- | --- | --- | --- | --- | --- | --- | --- |
|  | **MLL vs. control** | **AT1 vs. control** | **MLL vs. AT1** | **MLL vs. control** | **AT1 vs. control** | **MLL vs. AT1** | **MLL vs. control** | **AT1 vs. control** | **MLL vs. AT1** |
| **Proliferation of cells** | 5.9 | 4.8 | 2.5 | 2.88E-41 | 1.24E-43 | 6.07E-37 | 2374 (47/37/16) | 2209 (46/39/15) | 1430 (46/40/14) |
| **Cell proliferation of tumor cell lines** | 4.2 | 4.3 | 2.2 | 1.84E-16 | 8.70E-16 | 5.36E-15 | 1019 (51/40/9) | 940 (52/40/9) | 617 (49/42/9) |
| **Proliferation of blood cells** | 2.6 | 3.1 | -1.9 | 2.89E-17 | 9.62E-29 | 3.70E-14 | 557 (48/39/13) | 560 (49/40/11) | 342 (40/48/12) |
| **Cell survival** | 3.8 | 4.1 | -1.2 | 1.30E-20 | 1.83E-20 | 3.49E-15 | 930 (50/38/12) | 863 (50/38/12) | 554 (43/46/11) |
| **Cell death** | -0.5 | 0.5 | -2.2 | 4.50E-31 | 1.84E-32 | 5.11E-18 | 2149 (44/44/12) | 1995 (45/43/12) | 1231 (42/46/12) |
| **Cell movement** | 5.4 | 7.4 | -3.5 | 2.52E-39 | 1.32E-53 | 5.66E-43 | 1455 (49/37/13) | 1407 (51/35/14) | 928 (40/49/12) |
| **Chemotaxis** | 4.1 | 6.8 | -4.5 | 6.29E-20 | 4.33E-25 | 5.83E-19 | 381 (49/31/21) | 372 (55/25/20) | 249 (28/53/20) |
| **Recruitment of cells** | 3.9 | 5.9 | -5.0 | 4.54E-11 | 1.14E-18 | 4.05E-10 | 236 (57/34/8) | 243 (62/28/10) | 151 (25/66/9) |
| **Homing of cells** | 4.3 | 7.2 | -5.1 | 2.18E-21 | 4.05E-27 | 1.04E-18 | 405 (50/31/19) | 396 (56/25/19) | 260 (27/54/19) |
| **Angiogenesis** | 2.5 | 3.8 | -1.8 | 1.41E-18 | 4.98E-21 | 7.35E-22 | 640 (40/33/27) | 606 (42/30/28) | 418 (34/43/23) |

IPA core analyses were performed for each comparison (MLL vs. control, AT1 vs. control, and MLL vs. AT1). DEGs with FC ≥ 1.5 and p ≤ 0.05 were included in the analyses. Selected disease and function annotations from the downstream effect analysis are listed in the table. MLL, n = 7; AT1, n = 8; control, n = 8. GO, Gene ontology; IPA, Ingenuity Pathway Analysis
