# Supplementary material for: Aggressive rat prostate tumors reprogram the benign parts of the prostate and regional lymph nodes prior to metastasis
Source: PLoS One. 2017 May 4;12(5):e0176679. doi: 10.1371/journal.pone.0176679 (PMC5417597; doi:10.1371/journal.pone.0176679)
Supplement: S4 Table — A) TINT—Top 50 DEGs. B) TINT—Top 50 DEGs with a signal intensity value ≥ 500. C) TINT—Top 25 most highly expressed DEGs. DEG, Differentially expressed gene (FC ≥ 1.25, p ≤ 0.05); FC, Fold change; TINT, Tumor Instructed Normal Tissue. (DOCX) [file pone.0176679.s007.docx]

**S4 table. A)** **TINT - Top 50 DEGs**

| **MLL-TINT vs. control-prostate** | | **AT1-TINT vs. control-prostate** | | **MLL-TINT vs. AT1-TINT** | |
| --- | --- | --- | --- | --- | --- |
| **Gene** **symbol** | **FC** | **Gene symbol** | **FC** | **Gene symbol** | **FC** |
| **Upregulated genes** | | | | | |
| Hmox1 | 4.9 | Hmox1 | 2.4 | Slc30a2 | 8.9 |
| Ptx3 | 2.9 | ND6 | 2.0 | Grhl3 | 5.3 |
| Ubd | 2.8 | Ifi27 | 1.9 | Ubd | 4.7 |
| Usp18 | 2.8 | Fcn1 | 1.8 | Il12rb2 | 4.4 |
| Oas1b | 2.6 | Fam148a | 1.7 | Plau | 4.3 |
| Fcnb | 2.5 | Apof | 1.6 | Clu | 4.0 |
| Oas1i | 2.4 | Mc5r | 1.6 | Adcy2 | 3.3 |
| Ccl2 | 2.4 | Fam148b | 1.6 | Efna5 | 3.1 |
| Plau | 2.4 | Oas1i | 1.6 | Gstm6l | 3.0 |
| Oas1a | 2.4 | Siglec1 | 1.6 | Egln3 | 3.0 |
| Neurl3 | 2.3 | C1qa | 1.6 | Edn1 | 2.9 |
| Slc11a1 | 2.3 | Krtap9-1 | 1.6 | Serpine1 | 2.8 |
| Apol9a | 2.2 | Apol9a | 1.6 | Vtcn1 | 2.7 |
| Ms4a11 | 2.2 | Terc | 1.5 | Mmp15 | 2.6 |
| Ifi27l2b | 2.2 | Svep1 | 1.5 | Aldh1a3 | 2.6 |
| Ifi27 | 2.1 | Ugt2b34 | 1.5 | Sulf1 | 2.5 |
| Sell | 2.1 | Fgl1 | 1.5 | Mmp7 | 2.5 |
| Rsad2 | 2.0 | LOC100361180 | 1.5 | LOC685385 | 2.5 |
| Irf9 | 2.0 | LOC100363145 | 1.5 | Foxq1 | 2.5 |
| LOC290595 | 2.0 | Sfrp2 | 1.5 | Krt7 | 2.4 |
| Gpnmb | 2.0 | LOC64038 | 1.5 | Neurl3 | 2.4 |
| Ifit1 | 2.0 | Cd163 | 1.5 | Pon3 | 2.3 |
| Cd200r1 | 2.0 | Stt3b | 1.5 | Plk2 | 2.3 |
| Il8rb | 1.9 | Imp3 | 1.5 | Zmynd15 | 2.3 |
| Figf | 1.9 | Chp2 | 1.5 | Il1rn | 2.3 |
| **Downregulated genes** | | | | | |
| LOC100364156 | -3.7 | Mmp7 | -7.7 | LOC100364156 | -2.6 |
| Nkx3-1 | -3.5 | Slc30a2 | -6.0 | Ugt2b34 | -2.5 |
| Mmp7 | -3.1 | Grhl3 | -4.1 | Dok6 | -2.1 |
| Mia | -2.9 | Gp2 | -3.8 | ND6 | -2.0 |
| Cldn10 | -2.8 | RGD1559884 | -3.7 | Fut2 | -2.0 |
| Gp2 | -2.5 | Vtcn1 | -3.6 | Tfrc | -1.9 |
| Cnih2 | -2.4 | LOC363060 | -3.3 | Fgl1 | -1.9 |
| Fam3d | -2.2 | Clu | -3.2 | Klk1b21 | -1.8 |
| Pbk | -2.1 | Ceacam1 | -2.9 | Scd1 | -1.8 |
| Has2 | -2.0 | Capn13 | -2.8 | Olr659 | -1.7 |
| Myoc | -1.9 | Il12rb2 | -2.6 | Nppc | -1.7 |
| Ccnb2 | -1.9 | Pon3 | -2.6 | Krtap9-1 | -1.7 |
| Nppc | -1.8 | Adcy2 | -2.4 | Apof | -1.7 |
| Rnase2 | -1.8 | Foxq1 | -2.4 | Chdh | -1.7 |
| Upk1a | -1.8 | Krt23 | -2.4 | Lgals7 | -1.6 |
| Tfrc | -1.7 | LOC685385 | -2.4 | Mir200c | -1.6 |
| Ckap2 | -1.7 | Krt7 | -2.3 | Senp17 | -1.6 |
| Dok6 | -1.7 | Msln | -2.2 | Dbp | -1.6 |
| Cyss | -1.7 | Mpz | -2.2 | Slc7a11 | -1.6 |
| Fxyd3 | -1.7 | Efna5 | -2.2 | Slc17a7 | -1.6 |
| Nefl | -1.7 | Fam75a4 | -2.2 | Map2 | -1.6 |
| Pi15 | -1.7 | Sel1l3 | -2.2 | Tbxa2r | -1.6 |
| Mpz | -1.7 | LOC100360880 | -2.1 | Mthfd2 | -1.6 |
| Dbp | -1.7 | Slc39a8 | -2.1 | Bche | -1.6 |
| LOC684289 | -1.7 | Upk1a | -2.1 | Dsc3 | -1.6 |

DEG, Differentially expressed gene (FC ≥ 1.25, p ≤ 0.05); FC, Fold Change; TINT, Tumor Instructed Normal Tissue

**S4 Table. B) TINT – top 50 DEGs with a signal intensity value ≥ 500**

| **MLL-TINT vs. control-prostate** | | **AT1-TINT vs. control-prostate** | | **MLL-TINT vs. AT1-TINT** | |
| --- | --- | --- | --- | --- | --- |
| **Gene** **symbol** | **FC** | **Gene symbol** | **FC** | **Gene symbol** | **FC** |
| **Upregulated genes** | | | | | |
| Usp18 | 2.8 | ND6 | 2.0 | Plau | 4.3 |
| Oas1i | 2.4 | Ifi27 | 1.9 | Clu | 4.0 |
| Ccl2 | 2.4 | Fam148a | 1.7 | Efna5 | 3.1 |
| Plau | 2.4 | Fgl1 | 1.5 | Gstm6l | 3.0 |
| Ifi27 | 2.1 | Imp3 | 1.5 | Mmp15 | 2.6 |
| Ctgf | 1.6 | Sfrs5 | 1.4 | Sulf1 | 2.5 |
| RT1-S3 | 1.6 | Rps27a | 1.4 | Bace2 | 2.2 |
| Ddx58 | 1.6 | Ndufa6 | 1.4 | Krt15 | 2.1 |
| Gstm6l | 1.5 | Junb | 1.4 | Gstm7 | 2.1 |
| Mx2 | 1.5 | Arpc5l | 1.4 | Parm1 | 2.1 |
| Sfrs5 | 1.5 | Dusp1 | 1.3 | S100a11 | 2.1 |
| C4-2 | 1.5 | LOC690344 | 1.3 | Ctgf | 2.1 |
| Tnfrsf11b | 1.5 | Ldoc1 | 1.3 | Krt19 | 2.0 |
| Clec2g | 1.4 | Lama5 | 1.3 | Cyr61 | 2.0 |
| Gstm7 | 1.4 | Ddrgk1 | 1.3 | Gadd45b | 1.9 |
| Lima1 | 1.4 | Cyp7b1 | 1.3 | Clec2g | 1.9 |
| Fgl2 | 1.4 | Hnrnpa3 | 1.3 | Usp18 | 1.9 |
| Rnf39 | 1.4 | Sels | 1.3 | Anxa2 | 1.8 |
| Gadd45b | 1.4 | Chac2 | 1.3 | Cxcl16 | 1.8 |
| Efna5 | 1.4 | Hist1h2ail | 1.3 | Anxa1 | 1.8 |
| Cybb | 1.4 | Hk2 | 1.3 | Tacstd2 | 1.8 |
| Fcer1g | 1.4 | Bola1 | 1.3 | Wfdc2 | 1.7 |
| Junb | 1.4 | Aacs | 1.3 | Egr1 | 1.7 |
| Krt19 | 1.4 | Snx11 | 1.3 | Tpm1 | 1.7 |
| Rnf213 | 1.4 | Cbx4 | 1.3 | Sdc1 | 1.6 |
| **Downregulated genes** | | | | | |
| Dbp | -1.7 | Clu | -3.2 | ND6 | -2.0 |
| Klk1b21 | -1.6 | Ceacam1 | -2.9 | Fgl1 | -1.9 |
| RGD1359529 | -1.5 | S100a11 | -2.1 | Klk1b21 | -1.8 |
| Morc4 | -1.5 | Mmp15 | -2.0 | Dbp | -1.6 |
| Adh1 | -1.4 | Gstm6l | -2.0 | Mthfd2 | -1.6 |
| Lsm3 | -1.4 | Wfdc2 | -1.9 | Elovl6 | -1.5 |
| Mgst3 | -1.4 | Errfi1 | -1.9 | Ldoc1 | -1.5 |
| Elovl6 | -1.3 | Bace2 | -1.8 | Paqr5 | -1.5 |
| LOC100359977 | -1.3 | Egr1 | -1.8 | Morc4 | -1.5 |
| Angptl2 | -1.3 | Krt15 | -1.8 | Cyp7b1 | -1.5 |
| Acat2 | -1.3 | Parm1 | -1.6 | Tm7sf2 | -1.5 |
| RGD1565775 | -1.3 | Ano1 | -1.6 | Fam55c | -1.5 |
| H2afz | -1.3 | Lsm3 | -1.6 | Fam148a | -1.5 |
| Pycr1 | -1.3 | Anxa2 | -1.5 | Pdzk1ip1 | -1.4 |
| Mthfd2 | -1.3 | Igfbp5 | -1.5 | Aacs | -1.4 |
| Clk1 | -1.3 | Atp2b4 | -1.5 | Mrps12 | -1.4 |
| Rangap1 | -1.3 | Wif1 | -1.5 | Hk2 | -1.4 |
| Mrpl34 | -1.3 | Srd5a2 | -1.5 | RGD1561113 | -1.4 |
| Tm7sf2 | -1.3 | Clk1 | -1.5 | Prom2 | -1.4 |
| Slc35a2 | -1.3 | Krt19 | -1.5 | Rcan3 | -1.4 |
| Cuta | -1.3 | Gstm7 | -1.5 | LOC100360074 | -1.4 |
| Impa1 | -1.3 | Fam129a | -1.5 | Cst6 | -1.4 |
| Tmem184c | -1.3 | Sparcl1 | -1.4 | Hsd17b7 | -1.4 |
| Cbr1 | -1.3 | Timp3 | -1.4 | Cd38 | -1.4 |
| - | - | Adh1 | -1.4 | Herpud1 | -1.4 |

DEG, Differentially expressed gene (FC ≥ 1.25, p ≤ 0.05); FC, Fold Change; TINT, Tumor Instructed Normal Tissue

**S4 Table. C)** **TINT – top 25 most highly expressed DEGs**

| **MLL-TINT vs. control-prostate** | | | | **AT1-TINT vs. control-prostate** | | | | **MLL-TINT vs. AT1-TINT** | | | |
| --- | --- | --- | --- | --- | --- | --- | --- | --- | --- | --- | --- |
| **Gene** | **Intensity MLL** | **Intensity control** | **FC** | **Gene** | **Intensity AT1** | **Intensity control** | **FC** | **Gene** | **Intensity MLL** | **Intensity AT1** | **FC** |
| Cldn4 | 5151 | 4100 | 1.3 | Cyp7b1 | 3900 | 2964 | 1.3 | LOC310926 | 4925 | 6369 | -1.3 |
| Klk1b21 | 2938 | 4819 | -1.6 | Fam148a | 3735 | 2173 | 1.7 | Klk1b21 | 2938 | 5379 | -1.8 |
| Gstm7 | 3836 | 2671 | 1.4 | Ldoc1 | 3638 | 2732 | 1.3 | Clu | 4380 | 1086 | 4.0 |
| Krt19 | 3523 | 2536 | 1.4 | Clu | 1086 | 3438 | -3.2 | Insig1 | 3100 | 4078 | -1.3 |
| Gstm6l | 2765 | 1840 | 1.5 | Krt15 | 1606 | 2852 | -1.8 | Cyp7b1 | 2654 | 3900 | -1.5 |
| Tacstd2 | 2663 | 2073 | 1.3 | Gstm7 | 1833 | 2671 | -1.5 | Gstm7 | 3836 | 1833 | 2.1 |
| Ifi27 | 2656 | 1251 | 2.1 | Sparcl1 | 1838 | 2644 | -1.4 | Fam148a | 2564 | 3735 | -1.5 |
| Parm1 | 2438 | 1905 | 1.3 | Igfbp5 | 1696 | 2561 | -1.5 | Ldoc1 | 2448 | 3638 | -1.5 |
| Gas6 | 2261 | 1729 | 1.3 | Krt19 | 1726 | 2536 | -1.5 | Krt19 | 3523 | 1726 | 2.0 |
| Lyz2 | 2259 | 1743 | 1.3 | Psme2 | 1758 | 2241 | -1.3 | Krt15 | 3398 | 1606 | 2.1 |
| Ifngr1 | 2050 | 1614 | 1.3 | Tpm1 | 1566 | 2217 | -1.4 | Sec61b | 2441 | 3089 | -1.3 |
| Clec2g | 1932 | 1340 | 1.4 | Actg2 | 1676 | 2202 | -1.3 | Rcn2 | 2227 | 2816 | -1.3 |
| Rgs2 | 1798 | 1392 | 1.3 | Tacstd2 | 1517 | 2073 | -1.4 | Gstm6l | 2765 | 909 | 3.0 |
| Zfp36l1 | 1763 | 1398 | 1.3 | Cnn1 | 1545 | 2034 | -1.3 | Sec61g | 2008 | 2704 | -1.3 |
| Cxcl16 | 1619 | 1245 | 1.3 | Parm1 | 1165 | 1905 | -1.6 | Tacstd2 | 2663 | 1517 | 1.8 |
| Rnf213 | 1610 | 1265 | 1.3 | App | 1504 | 1898 | -1.3 | Tpm1 | 2615 | 1566 | 1.7 |
| Imp3 | 1609 | 1269 | 1.3 | Gstm6l | 909 | 1840 | -2.0 | Sparcl1 | 2546 | 1838 | 1.4 |
| Lima1 | 1546 | 1080 | 1.4 | Ifngr1 | 1281 | 1614 | -1.3 | Cd14 | 2504 | 1912 | 1.3 |
| Rnf39 | 1478 | 1037 | 1.4 | Anxa1 | 1129 | 1583 | -1.4 | Acta1 | 2481 | 1841 | 1.3 |
| Elovl6 | 1097 | 1463 | -1.3 | Cxcl14 | 1178 | 1538 | -1.3 | Parm1 | 2438 | 1165 | 2.1 |
| Acat2 | 1070 | 1414 | -1.3 | Ctsh | 1205 | 1531 | -1.3 | Igfbp5 | 2404 | 1696 | 1.4 |
| Trim25 | 1379 | 1086 | 1.3 | Myh11 | 1125 | 1503 | -1.3 | Des | 2259 | 1777 | 1.3 |
| Impa1 | 1080 | 1356 | -1.3 | Fbln1 | 1070 | 1470 | -1.4 | Actg2 | 2221 | 1676 | 1.3 |
| LOC100359977 | 997 | 1323 | -1.3 | Flna | 1009 | 1380 | -1.4 | Psme2 | 2199 | 1758 | 1.3 |
| MGC72974 | 1301 | 1031 | 1.3 | Sesn3 | 1019 | 1377 | -1.4 | Myl12b | 2188 | 1712 | 1.3 |

DEG, Differentially expressed gene (FC ≥ 1.25, p ≤ 0.05); FC, Fold Change; TINT, Tumor Instructed Normal Tissue

**Comments to S4 table**: Among top factors upregulated in AT1-TINT vs. control was Hmox-1 (see below), Ifi27 - an interferon-alpha induced factor that promotes cytotoxic T-cells [1], and Siglec1 (Cd169) - a factor expressed in antigen-presenting cells [2]. Among top factors downregulated in AT1-TINT vs. control was Grhl3 - a factor that promotes cell migration and invasion [3], Vtcn1 - a factor that restricts antitumor T-cell responses and is associated with metastasis [4], Slc30a2 - an epithelial Zn-transporter shown to be central for prostate function [5], and the immune-response and apoptosis regating factor Clu [6]. Among top factors upregulated in MLL-TINT vs. control prostate we find for example Hmox1 - a macrophage-associated factor that promotes metastasis [7], Ptx3 - a factor that promotes immune-escape and is associated with prostate cancer progression [8], Usp18 - a factor that stimulates immune responses and increase EGFR signaling [9], Ubd - a factor that promotes tumor growth and regulates immune responses [10], Plau a factor that promotes invasion and metastasis [11], Ccl2 - a macrophage-attracting factor [12], Oas1 - a macrophage derived factor that promotes innate immune responses [13], Figf (Vegfd) - a lymphangiogenic factor [14], and the tumor promoting stroma factor Ctgf [15]. Among top factors downregulated in MLL-TINT was the prostate epithelial cell specific transcription factor Nkx3.1 [16] (Nkx3.1 was also decreased in AT1-TINT but here this change was not statistically significant).

**References S4 table**:

1. Wenzel J, Tomiuk S, Zahn S, Kusters D, Vahsen A, Wiechert A, et al. Transcriptional profiling identifies an interferon-associated host immune response in invasive squamous cell carcinoma of the skin. Int J Cancer. 2008;123(11):2605-15. doi: 10.1002/ijc.23799. PubMed PMID: 18770863.

2. O'Neill AS, van den Berg TK, Mullen GE. Sialoadhesin - a macrophage-restricted marker of immunoregulation and inflammation. Immunology. 2013;138(3):198-207. doi: 10.1111/imm.12042. PubMed PMID: 23181380; PubMed Central PMCID: PMCPMC3573273.

3. Zhao P, Guo S, Tu Z, Di L, Zha X, Zhou H, et al. Grhl3 induces human epithelial tumor cell migration and invasion via downregulation of E-cadherin. Acta Biochim Biophys Sin (Shanghai). 2016;48(3):266-74. doi: 10.1093/abbs/gmw001. PubMed PMID: 26837418; PubMed Central PMCID: PMCPMC4885135.

4. Zang X, Thompson RH, Al-Ahmadie HA, Serio AM, Reuter VE, Eastham JA, et al. B7-H3 and B7x are highly expressed in human prostate cancer and associated with disease spread and poor outcome. Proc Natl Acad Sci U S A. 2007;104(49):19458-63. doi: 10.1073/pnas.0709802104. PubMed PMID: 18042703; PubMed Central PMCID: PMCPMC2148311.

5. Iguchi K, Morihara N, Usui S, Hayama M, Sugimura Y, Hirano K. Castration- and aging-induced changes in the expression of zinc transporter and metallothionein in rat prostate. J Androl. 2011;32(2):144-50. doi: 10.2164/jandrol.110.011205. PubMed PMID: 20798384.

6. Koltai T. Clusterin: a key player in cancer chemoresistance and its inhibition. Onco Targets Ther. 2014;7:447-56. doi: 10.2147/OTT.S58622. PubMed PMID: 24672247; PubMed Central PMCID: PMCPMC3964162.

7. Halin Bergstrom S, Nilsson M, Adamo H, Thysell E, Jernberg E, Stattin P, et al. Extratumoral Heme Oxygenase-1 (HO-1) Expressing Macrophages Likely Promote Primary and Metastatic Prostate Tumor Growth. PLoS One. 2016;11(6):e0157280. doi: 10.1371/journal.pone.0157280. PubMed PMID: 27280718; PubMed Central PMCID: PMCPMC4900522.

8. Stallone G, Cormio L, Netti GS, Infante B, Selvaggio O, Fino GD, et al. Pentraxin 3: a novel biomarker for predicting progression from prostatic inflammation to prostate cancer. Cancer Res. 2014;74(16):4230-8. doi: 10.1158/0008-5472.CAN-14-0369. PubMed PMID: 24950910.

9. Hong B, Li H, Lu Y, Zhang M, Zheng Y, Qian J, et al. USP18 is crucial for IFN-gamma-mediated inhibition of B16 melanoma tumorigenesis and antitumor immunity. Mol Cancer. 2014;13:132. doi: 10.1186/1476-4598-13-132. PubMed PMID: 24884733; PubMed Central PMCID: PMCPMC4057584.

10. Aichem A, Groettrup M. The ubiquitin-like modifier FAT10 in cancer development. Int J Biochem Cell Biol. 2016;79:451-61. doi: 10.1016/j.biocel.2016.07.001. PubMed PMID: 27393295.

11. McMahon BJ, Kwaan HC. Components of the Plasminogen-Plasmin System as Biologic Markers for Cancer. Adv Exp Med Biol. 2015;867:145-56. doi: 10.1007/978-94-017-7215-0_10. PubMed PMID: 26530365.

12. Zhang J, Lu Y, Pienta KJ. Multiple roles of chemokine (C-C motif) ligand 2 in promoting prostate cancer growth. J Natl Cancer Inst. 2010;102(8):522-8. doi: 10.1093/jnci/djq044. PubMed PMID: 20233997; PubMed Central PMCID: PMCPMC2857800.

13. Kazma R, Mefford JA, Cheng I, Plummer SJ, Levin AM, Rybicki BA, et al. Association of the innate immunity and inflammation pathway with advanced prostate cancer risk. PLoS One. 2012;7(12):e51680. doi: 10.1371/journal.pone.0051680. PubMed PMID: 23272139; PubMed Central PMCID: PMCPMC3522730.

14. Woollard DJ, Opeskin K, Coso S, Wu D, Baldwin ME, Williams ED. Differential expression of VEGF ligands and receptors in prostate cancer. Prostate. 2013;73(6):563-72. doi: 10.1002/pros.22596. PubMed PMID: 23038639.

15. Yang F, Tuxhorn JA, Ressler SJ, McAlhany SJ, Dang TD, Rowley DR. Stromal expression of connective tissue growth factor promotes angiogenesis and prostate cancer tumorigenesis. Cancer Res. 2005;65(19):8887-95. doi: 10.1158/0008-5472.CAN-05-1702. PubMed PMID: 16204060.

16. Abate-Shen C, Shen MM, Gelmann E. Integrating differentiation and cancer: the Nkx3.1 homeobox gene in prostate organogenesis and carcinogenesis. Differentiation. 2008;76(6):717-27. doi: 10.1111/j.1432-0436.2008.00292.x. PubMed PMID: 18557759; PubMed Central PMCID: PMCPMC3683569.
