# Supplementary material for: Aggressive rat prostate tumors reprogram the benign parts of the prostate and regional lymph nodes prior to metastasis
Source: PLoS One. 2017 May 4;12(5):e0176679. doi: 10.1371/journal.pone.0176679 (PMC5417597; doi:10.1371/journal.pone.0176679)
Supplement: S6 Table — A) LN—Top 50 DEGs. B) LN—Top 50 DEGs with a signal intensity value ≥ 500. C) LN—Top 25 most highly expressed DEGs. DEG, Differentially expressed gene (FC ≥ 1.25, p ≤ 0.05); FC, Fold change; LN, Lymph Node. (DOCX) [file pone.0176679.s009.docx]

**S6 Table. A) LN - Top 50 DEGs**

| **MLL-LN vs. control-LN** | | **AT1-LN vs. control-LN** | | **MLL-LN vs. AT1-LN** | |
| --- | --- | --- | --- | --- | --- |
| **Gene** **symbol** | **FC** | **Gene symbol** | **FC** | **Gene symbol** | **FC** |
| **Upregulated genes** | | | | | |
| RGD1561247 | 6.8 | LOC100364840 | 10.4 | LOC690359 | 2.4 |
| Il21 | 4.5 | Ighg | 4.0 | Il1r2 | 2.2 |
| aicda | 4.4 | LOC100362601 | 3.2 | RGD1310335 | 2.0 |
| LOC500159 | 4.4 | Ns5atp9 | 2.9 | Sgol2 | 1.9 |
| Cdk1 | 4.3 | Fgf23 | 2.9 | Cenpi | 1.9 |
| Rrm2 | 4.3 | Rrm2 | 2.8 | Exo1 | 1.8 |
| Bub1 | 4.3 | Tnfrsf4 | 2.7 | Gpr55 | 1.8 |
| Mybl2 | 4.3 | LOC680311 | 2.7 | Ttk | 1.8 |
| Irg1 | 4.2 | Mybl2 | 2.7 | Kif23 | 1.8 |
| RGD1562099 | 4.1 | Vsig4 | 2.7 | Cep72 | 1.7 |
| Ska3 | 4.1 | Plk1 | 2.6 | Chek1 | 1.7 |
| Ccne1 | 4.1 | Cdc20 | 2.6 | Melk | 1.7 |
| Cep55 | 4.1 | Cdk1 | 2.6 | Ctla4 | 1.7 |
| Cdkn3 | 4.0 | Mef2b | 2.5 | Mastl | 1.7 |
| Pbk | 4.0 | Nusap1 | 2.5 | Mir130b | 1.6 |
| Cenpk | 4.0 | Nek2 | 2.5 | Kif20b | 1.6 |
| Cdc20 | 3.9 | Ccnb2 | 2.5 | Mad2l1 | 1.6 |
| Tmem97 | 3.9 | LOC690044 | 2.5 | Hspa13 | 1.6 |
| Nusap1 | 3.8 | Cenpt | 2.5 | Pla2g7 | 1.6 |
| Dhfr | 3.8 | Hist1h2bc | 2.5 | Fancb | 1.6 |
| Cenpt | 3.8 | Cenpk | 2.5 | Dnajb11 | 1.6 |
| Ect2 | 3.8 | Kif18b | 2.4 | Polq | 1.6 |
| Nek2 | 3.8 | Cdc6 | 2.4 | Cep76 | 1.6 |
| Dtl | 3.7 | Cdkn3 | 2.4 | LOC683179 | 1.6 |
| Sgol1 | 3.7 | Ube2c | 2.4 | Fut8 | 1.6 |
| **Downregulated genes** | | | | | |
| Prg4 | -8.3 | Prg4 | -13.6 | C1ql2 | -2.0 |
| Retnlg | -6.6 | C6 | -4.4 | Clec1b | -2.0 |
| C6 | -3.6 | Fn1 | -3.1 | Nr1d1 | -1.7 |
| Tgfb2 | -3.2 | Mrgprx2 | -2.4 | Klf9 | -1.6 |
| F13a1 | -2.7 | Alox15 | -2.3 | Rps27 | -1.6 |
| Mcpt1 | -2.7 | Olr1058 | -2.2 | Fxyd2 | -1.6 |
| Mcpt2 | -2.5 | Emr4 | -2.1 | LOC688582 | -1.6 |
| Cma1 | -2.5 | F5 | -2.1 | Clec1a | -1.6 |
| Cpa3 | -2.5 | Olr1585 | -2.1 | Cyp7b1 | -1.6 |
| LOC688582 | -2.3 | N5 | -1.9 | Csf1 | -1.6 |
| F5 | -2.2 | Mcpt2 | -1.9 | Tgfbr3 | -1.6 |
| Nrxn1 | -2.2 | Car5b | -1.9 | Ccbe1 | -1.6 |
| LOC100363145 | -2.1 | Scn7a | -1.9 | Wbscr17 | -1.5 |
| Tpsb2 | -2.1 | Il1rl1 | -1.9 | Gprc5c | -1.5 |
| RGD1565804 | -2.1 | RGD1565804 | -1.8 | Gzmm | -1.5 |
| Tsc22d3 | -2.0 | Hdc | -1.8 | Gpr68 | -1.5 |
| Ahnak | -2.0 | Aox3 | -1.7 | Gfra2 | -1.5 |
| Tgfbr3 | -2.0 | Syne2 | -1.7 | Epm2a | -1.5 |
| Hdc | -1.9 | Pot1b | -1.7 | Tgfbi | -1.5 |
| Nr1d1 | -1.9 | Tsc22d3 | -1.7 | Snx33 | -1.4 |
| Sult1a1 | -1.9 | Cpa3 | -1.7 | Igfbp3 | -1.4 |
| Errfi1 | -1.9 | Errfi1 | -1.7 | Dbp | -1.4 |
| Reln | -1.9 | Tpsb2 | -1.7 | Fam13c1 | -1.4 |
| Scn7a | -1.9 | Macf1 | -1.7 | Garnl1 | -1.4 |
| Mir505 | -1.9 | Ahnak | -1.7 | Srgap3 | -1.4 |

DEG, Differentially expressed gene (FC ≥ 1.25, p ≤ 0.05); FC, Fold change; LN, Lymph node

**S6 Table. B) LN – top 50 DEGs with a signal intensity value ≥ 500**

| **MLL-LN vs. control-LN** | | **AT1-LN vs. control-LN** | | **MLL-LN vs. AT1-LN** | |
| --- | --- | --- | --- | --- | --- |
| **Gene** **symbol** | **FC** | **Gene symbol** | **FC** | **Gene symbol** | **FC** |
| **Upregulated genes** | | | | | |
| RGD1561247 | 6.8 | Ns5atp9 | 2.9 | Dnajb11 | 1.6 |
| LOC500159 | 4.4 | Rrm2 | 2.8 | Fut8 | 1.6 |
| Rrm2 | 4.3 | Tnfrsf4 | 2.7 | Slc35b1 | 1.5 |
| Ska3 | 4.1 | Plk1 | 2.6 | Nipal1 | 1.5 |
| Cdkn3 | 4.0 | Cdc20 | 2.6 | Uba5 | 1.5 |
| Cdc20 | 3.9 | LOC690044 | 2.5 | Spcs3 | 1.5 |
| Tmem97 | 3.9 | Hist1h2ak | 2.3 | Whsc1 | 1.5 |
| Plk1 | 3.7 | Igh-6 | 2.2 | Hat1 | 1.4 |
| Ns5atp9 | 3.5 | Hist1h2bf | 2.2 | Ssr3 | 1.4 |
| Pttg1 | 3.5 | LOC366766 | 2.1 | Edem1 | 1.4 |
| Ccnf | 3.5 | Hist1h1a | 2.1 | Ak2 | 1.4 |
| Igh-6 | 3.5 | Hist1h2bl | 2.1 | Odc1 | 1.4 |
| Tpx2 | 3.5 | RGD1310251 | 2.1 | Nat13 | 1.4 |
| Fbxo5 | 3.5 | Aurkb | 2.0 | Arrdc3 | 1.4 |
| Ccna2 | 3.4 | Hist1h1b | 2.0 | Slc16a6 | 1.4 |
| Tnfrsf4 | 3.4 | Top2a | 2.0 | Dpagt1 | 1.4 |
| LOC366772 | 3.3 | Mki67 | 2.0 | Xrcc6 | 1.4 |
| Derl3 | 3.3 | Dctpp1 | 2.0 | Slc30a6 | 1.4 |
| Creld2 | 3.3 | Cks1b | 1.9 | Prps1 | 1.4 |
| Asf1b | 3.1 | Psat1 | 1.9 | Erp44 | 1.4 |
| RGD1310251 | 3.1 | Isg20 | 1.8 | Guf1 | 1.4 |
| Uhrf1 | 3.0 | Igha | 1.8 | Azin1 | 1.3 |
| Top2a | 2.9 | Hist2h4 | 1.7 | Rcbtb2 | 1.3 |
| Aurkb | 2.9 | RGD1564767 | 1.7 | Gtf2a1 | 1.3 |
| Hist1h1a | 2.7 | Hist1h2bb | 1.7 | Xpo1 | 1.3 |
| **Downregulated genes** | | | | | |
| Prg4 | -8.3 | Prg4 | -13.6 | Fxyd2 | -1.6 |
| Mcpt1 | -2.7 | Fn1 | -3.1 | Tgfbr3 | -1.6 |
| Cpa3 | -2.5 | Tsc22d3 | -1.7 | Gzmm | -1.5 |
| Mcpt2 | -2.5 | Cpa3 | -1.7 | Gfra2 | -1.5 |
| Tsc22d3 | -2.0 | Errfi1 | -1.7 | Tgfbi | -1.5 |
| Tgfbr3 | -2.0 | Macf1 | -1.7 | Snx33 | -1.4 |
| Errfi1 | -1.9 | Mcpt1 | -1.7 | Igfbp3 | -1.4 |
| Lrp1 | -1.8 | Rnf213 | -1.6 | Dbp | -1.4 |
| Igfbp5 | -1.8 | Ccnl2 | -1.6 | Slco2b1 | -1.4 |
| Rnf213 | -1.8 | Utrn | -1.6 | Fgfr1 | -1.4 |
| RGD1359529 | -1.7 | Arrdc3 | -1.6 | Ctsl1 | -1.4 |
| Abcc9 | -1.7 | Lrp1 | -1.5 | Gpr146 | -1.4 |
| Siglec1 | -1.7 | Xkrx | -1.5 | Plvap | -1.3 |
| Lifr | -1.7 | Lyst | -1.5 | LOC100361180 | -1.3 |
| Ccl24 | -1.7 | Mcpt2 | -1.5 | Rps24 | -1.3 |
| Utrn | -1.7 | Akap13 | -1.5 | Hyal1 | -1.3 |
| Nr2f2 | -1.7 | Ccnt2 | -1.5 | Igfbp4 | -1.3 |
| Ltbp4 | -1.7 | Zcchc7 | -1.5 | Fbxl14 | -1.3 |
| Igfbp3 | -1.7 | RGD1359529 | -1.5 | Fam101b | -1.3 |
| Mrc1 | -1.7 | Znf292 | -1.5 | Nphp1 | -1.3 |
| Csf1 | -1.6 | Arl6ip1 | -1.5 | Bcl9l | -1.3 |
| Tns1 | -1.6 | Ccnl1 | -1.5 | Zbtb4 | -1.3 |
| Macf1 | -1.6 | Fzd4 | -1.5 | RGD1306119 | -1.3 |
| Tinagl1 | -1.6 | Tmem2 | -1.4 | RGD1565415 | -1.3 |
| Gsn | -1.6 | Ddr2 | -1.4 | Ubqln2 | -1.3 |

DEG, Differentially expressed gene (FC ≥ 1.25, p ≤ 0.05); FC, Fold change; LN, Lymph node

**S6 Table. C) LN – top 25 most highly expressed DEGs**

| **MLL-LN vs. control-LN** | | | | **AT1-LN vs. control-LN** | | | | **MLL-LN vs. AT1-LN** | | | |
| --- | --- | --- | --- | --- | --- | --- | --- | --- | --- | --- | --- |
| **Gene** | **Intensity MLL** | **Intensity control** | **FC** | **Gene** | **Intensity AT1** | **Intensity control** | **FC** | **Gene** | **Intensity MLL** | **Intensity AT1** | **FC** |
| Hist1h2bc | 7485 | 5386 | 1.4 | Hist1h2bc | 7460 | 5386 | 1.4 | Dad1 | 3860 | 3017 | 1.3 |
| IgG-2a | 7008 | 4257 | 1.6 | Hist1h2bn | 6694 | 4753 | 1.4 | Csf1r | 2189 | 2772 | -1.3 |
| Hist1h2bn | 6811 | 4753 | 1.4 | Hist1h2bl | 6665 | 4756 | 1.4 | Nat13 | 2553 | 1844 | 1.4 |
| Hist1h2bl | 6801 | 4756 | 1.4 | IgG-2a | 6332 | 4257 | 1.5 | Rps24 | 1647 | 2160 | -1.3 |
| Hist2h3c2 | 6774 | 3917 | 1.7 | LOC680322 | 6202 | 4010 | 1.5 | Edem1 | 1830 | 1290 | 1.4 |
| LOC680322 | 6553 | 4010 | 1.6 | RGD1564392 | 6083 | 4137 | 1.5 | Spcs3 | 1781 | 1196 | 1.5 |
| RGD1564392 | 6274 | 4137 | 1.5 | Hist2h3c2 | 5797 | 3917 | 1.5 | Dck | 1664 | 1264 | 1.3 |
| Hist1h2bh | 6022 | 4776 | 1.3 | Hist2h4 | 5475 | 3162 | 1.7 | Ssr1 | 1612 | 1252 | 1.3 |
| Hist1h2ail | 5623 | 3781 | 1.5 | Hist1h2ail | 5218 | 3781 | 1.4 | RGD1565415 | 1257 | 1602 | -1.3 |
| RGD1564284 | 5618 | 4301 | 1.3 | Hist2h2ab | 4656 | 3405 | 1.4 | Tgfbi | 1071 | 1571 | -1.5 |
| RGD1564318 | 5272 | 4040 | 1.3 | Txnip | 2894 | 4038 | -1.4 | Odc1 | 1557 | 1122 | 1.4 |
| Hist2h2ab | 5187 | 3491 | 1.5 | Psmb9 | 3986 | 3161 | 1.3 | Igfbp3 | 1071 | 1548 | -1.4 |
| Hist2h4 | 4974 | 3162 | 1.6 | Hist1h2bb | 3792 | 2256 | 1.7 | Clptm1l | 1497 | 1192 | 1.3 |
| Igha | 4777 | 2051 | 2.3 | Ccl19 | 3626 | 2843 | 1.3 | Paics | 1424 | 1131 | 1.3 |
| Hspa5 | 4467 | 3399 | 1.3 | Igha | 3603 | 2051 | 1.8 | Plvap | 1021 | 1373 | -1.3 |
| Txnip | 2866 | 4038 | -1.4 | Igh-6 | 3019 | 2254 | 1.3 | Sap18 | 1771 | 1369 | 1.3 |
| Sepp1 | 3098 | 3993 | -1.3 | Ndufa4 | 2983 | 2318 | 1.3 | Cbx5 | 1355 | 1062 | 1.3 |
| Psmb9 | 3961 | 3161 | 1.3 | Tmem93 | 2883 | 2299 | 1.3 | Slc35b1 | 1345 | 887 | 1.5 |
| Hist1h2bb | 3933 | 2256 | 1.7 | LOC679840 | 2607 | 1891 | 1.4 | Alg3 | 1337 | 1048 | 1.3 |
| Igh-6 | 3870 | 2254 | 1.7 | Psme2 | 2600 | 2026 | 1.3 | RGD1560888 | 1256 | 958 | 1.3 |
| Dad1 | 3860 | 2995 | 1.3 | Gpr174 | 2047 | 2569 | -1.3 | Uqcrfs1 | 1215 | 966 | 1.3 |
| Eno1 | 3735 | 2876 | 1.3 | Uqcr | 2552 | 1904 | 1.3 | Uba5 | 1140 | 756 | 1.5 |
| RGD1310251 | 3727 | 1208 | 3.1 | RGD1310251 | 2521 | 1208 | 2.1 | Fxyd2 | 674 | 1087 | -1.6 |
| Ndufc2 | 3674 | 2897 | 1.3 | S100a1 | 2490 | 1766 | 1.4 | Zmpste24 | 1069 | 832 | 1.3 |
| Ccl19 | 3637 | 2843 | 1.3 | Sptbn1 | 1798 | 2250 | -1.3 | RGD1565496 | 1066 | 829 | 1.3 |

DEG, Differentially expressed gene (FC ≥ 1.25, p ≤ 0.05); FC, Fold change; LN, Lymph node
