# Supplementary material for: Aggressive rat prostate tumors reprogram the benign parts of the prostate and regional lymph nodes prior to metastasis
Source: PLoS One. 2017 May 4;12(5):e0176679. doi: 10.1371/journal.pone.0176679 (PMC5417597; doi:10.1371/journal.pone.0176679)
Supplement: S1 Copyright Permission — (PDF) [file pone.0176679.s011.pdf]

S1 Copyright permission

QIAGEN Ingenuity product support

To whom this may concern:

Dr. Thysell has been granted permission by QIAGEN Silicon Valley to use copyrighted figures generated from Ingenuity Pathways Analysis in his/her publication. Figures produced from IPA are available under an open-access CC-BY license for purposes of publication.

If you have any further questions, please contact Ingenuity applications Customer Support at support-ingenuity@qiagen.com.

Best regards,

Jasmin

Dr. Jasmin Droege
Senior Scientist, Advanced Genomics Support
